# Hydrogen spillover as an interfacial strategy for nitrate electroreduction to ammonia

**DOI:** 10.1039/d6sc04282a

**Published:** 2026-07-16

**Authors:** Zhiwei Wang, Junlong Zheng, Xianghua Hou, Yinghong Wu, Longchao Zhuo, Wenxian Liu, Ligang Feng, Xijun Liu

**Affiliations:** a Guangxi Key Laboratory of Processing for Non-ferrous Metals and Featured Materials, MOE Key Laboratory of New Processing Technology for Nonferrous Metals and Materials, School of Resources, Environment and Materials, Guangxi University Nanning 530004 Guangxi China houxianghua191031@163.com yhwu@ipe.ac.cn xjliu@gxu.edu.cn; b Guangxi Technological College of Machinery and Electricity Nanning 530007 Guangxi China; c National Engineering Research Center of Green Recycling for Strategic Metal Resources, Institute of Process Engineering, Chinese Academy of Sciences Beijing 100190 China; d School of Materials Science and Engineering, Xi'an University of Technology Xi'an 710048 China; e College of Materials Science and Engineering, Zhejiang University of Technology Hangzhou 310014 Zhejiang China liuwx@zjut.edu.cn; f Faculty of Materials Science and Engineering, Kunming University of Science and Technology Kunming 650093 China

## Abstract

Nitrate electroreduction to ammonia (NRA) offers a route for ammonia recovery from nitrate-containing water streams while contributing to water remediation. However, its multi-step deoxygenation and hydrogenation pathway exhibits suboptimal efficiency. Rational electrocatalyst design is central to overcoming this bottleneck. Hydrogen spillover spatially decouples water-dissociation-driven H* supply from substrate hydrogenation, providing an interfacial strategy to balance activity and selectivity. This review methodically examines recent advancements in hydrogen-spillover-enhanced NRA. Catalyst design is discussed from five aspects: electronic structure engineering of Cu-based catalysts, donor and reduction phase coupling, H* migration direction control, migration pathway shortening, and interfacial microenvironment regulation. This comprehensive strategy is meticulously designed to enhance ammonia production rate and faradaic efficiency. Concurrently, we synthesize the principal characterization techniques currently deployed to probe hydrogen spillover and highlight their mechanistic diagnostic value. We conclude by identifying the outstanding challenges and future directions in this field.

## Abstract 2

Nitrate electroreduction to ammonia (NRA) offers a route for ammonia recovery from nitrate-containing water streams while contributing to water remediation.

## Graphical abstract

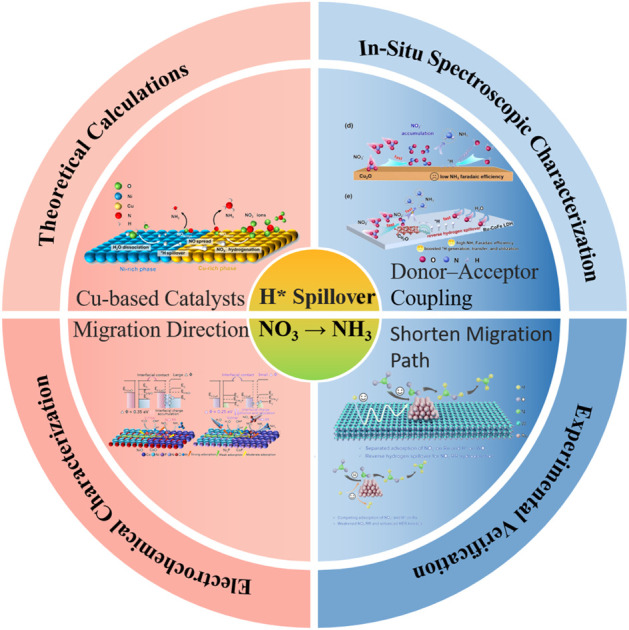

## 1. Introduction

Ammonia (NH_3_) is an indispensable chemical feedstock with global annual demand exceeding 150 million tons, underpinning food production and human sustenance.^1,2^ The predominant method for industrial ammonia synthesis is the Haber–Bosch process, which operates at elevated temperatures and pressures (400–500 °C, 150–300 bar). Although N_2_ hydrogenation to NH_3_ is exothermic at the reaction level, conventional NH_3_ manufacture remains energy and carbon intensive at the process level because of fossil-based H_2_ production, gas purification, compression, recycle operation, and heat management.^3–7^ This distinction is essential because NH_3_ is an energy carrier, and energy input is inherently required for its synthesis. Electrochemical processes that facilitate the reduction of nitrogen-containing small molecules to ammonia under mild conditions have thus garnered significant interest.^8–12^ A comparison of the nitrogen reduction reaction (NRR) reveals that its N≡N bond carries a formidable dissociation energy of 941 kJ mol^−1^. In contrast, nitrate (NO_3_^−^) offers a significantly lower N–O bond dissociation energy (204 kJ mol^−1^), high aqueous solubility, and favorable reduction kinetics. These properties position nitrate as a more viable substrate for electrochemical NH_3_ recovery, rather than a replacement feedstock for primary artificial nitrogen fixation.^13–17^ Fossil fuel combustion, excessive nitrogen fertilizer use, and untreated industrial wastewater discharge have steadily increased nitrate concentrations in surface water and groundwater.^18,19^ A substantial fraction of nitrate pollution is a downstream product of anthropogenic reactive nitrogen, including fertilizer-derived NH_3_ that has been oxidized, leached, and dispersed through agricultural water systems. The World Health Organization establishes a maximum acceptable level for NO_3_^−^ in drinking water at 50 mg L^−1^. This benchmark is frequently exceeded in numerous intensive agricultural regions globally.^20^ Excess nitrate accelerates eutrophication and degrades aquatic ecosystems. It also threatens human health through drinking water and the food chain.^21^ Consequently, the transformation of nitrate pollutants into valuable byproducts, such as ammonia, *via* the NRA process, holds both industrial and environmental significance.^22,23^ NRA utilizes water as the proton source and renewable electricity as the driving force, positioning it as a downstream nitrate abatement and reactive nitrogen recovery technology under mild electrochemical conditions.^24,25^ It should not be presented as a substitute for Haber–Bosch primary NH_3_ production from atmospheric N_2_. The integration of the NRA with plasma-driven nitrogen oxidation and ammonia fuel cells has the potential to enhance the efficacy of a closed-loop nitrogen cycle.^26–31^

It is important to note, however, that the NRA is a complex nine proton, eight electron transfer reaction. This process encompasses the adsorption and activation of NO_3_^−^, sequential N–O bond cleavage, and stepwise deoxygenation and hydrogenation of NO_*x*_ intermediates (NO_3_, NO_2_, NO, NHO, NH_2_OH, and NH_2_). These processes require active hydrogen (H*) derived from water dissociation.^32–35^ Among the aforementioned steps, the initial hydrogenation, NO → NHO, is identified by density functional theory (DFT) calculations as the rate-determining step with the highest energy barrier.^36^ This step is particularly sensitive to the instantaneous availability of surface H. In cases where H supply is inadequate, NO accumulates on the catalyst surface, impeding active-site regeneration and potentially inducing N–N coupling to form N_2_O or N_2_. This phenomenon can lead to a deterioration in NH_2_ selectivity. Conversely, excessive H coverage promotes the competing hydrogen evolution reaction (HER) *via* the Tafel or Heyrovsky pathway, thereby depressing faradaic efficiency (FE).^37,38^ In neutral or alkaline electrolytes, H* originates principally from the Volmer step (H_2_O + e^−^ → H*+ OH^−^). However, the sluggish kinetics of H–OH bond cleavage frequently leave the deep hydrogenation of NO_*X*_ intermediates starved of hydrogen.^39^ The fundamental contradiction inherent in this system stems from the fact that, in a conventional single-site catalyst, the generation of H* and NO_*x*_ hydrogenation occur at the same active center. Suppressing HER tends to result in a reduction in H* supply, while enhancing water dissociation accelerates H_2_ evolution.^40^ The development of electrocatalysts that spatially decouple H* provision from substrate hydrogenation is therefore essential for advancing NO_3_RR performance.^41^

H* refers to hydrogen generated by molecular hydrogen dissociation or water reduction at metal active sites. It can migrate to a support, an adjacent metal site, or another hydrogen-accepting component through surface diffusion or bulk transport. This interfacial migration was classically evidenced by Khoobiar using Pt–Al_2_O_3_ catalysts, where hydrogen atoms generated on Pt migrated to neighboring particles.^42^ In recent years, hydrogen spillover has demonstrated marked advantages across diverse electrocatalytic reactions in promoting proton adsorption, accelerating hydrogen desorption, and rerouting reaction pathways, establishing it as a key lever for catalytic performance enhancement.^43–45^ At its core, hydrogen spillover exemplifies multi-site cooperativity, whereby donor sites effectively dissociate water to yield H*, while acceptor sites adsorb, activate, and progressively hydrogenate the substrate. The interface or support between these sites provides a directional H* transport channel.^46^ A judicious tuning of the electronic structure and interfacial work function difference between the metal and support can lower the hydrogen migration barrier and accelerate reaction kinetics.^47^ In the context of the NRA, this spatial functional separation disrupts the inherent direct competition between the HER and NRA, which is characteristic of single-site catalysts. H* generation at donor sites occurs in a rapid manner, leading to its migration to NO_*x*_-rich reduction sites. At these sites, H* exhibits a preference for consumption in hydrogenation processes as opposed to accumulation and subsequent coupling at the donor surface.^48,49^ Despite the extensive documentation of hydrogen spillover, the mechanistic roles, regulatory principles, and characterization strategies of this phenomenon across various reaction systems remain incompletely resolved. Consequently, a systematic synthesis of the mechanism, application, and characterization of hydrogen spillover is timely and significant for deepening mechanistic understanding and guiding rational catalyst design.

The following structure has been employed in the organization of this review. It will first introduce the fundamental mechanism of hydrogen spillover. We then proceed to survey the latest advances in hydrogen-spillover-enhanced NRA. Subsequently, a systematic cataloging of the principal characterization methods and their respective strengths is undertaken. In conclusion, we identify the current research bottlenecks and outline future directions.

## 2. Mechanism

Hydrogen spillover is widely recognized as a significant class of interfacial dynamic behavior in catalysis. The term “H* migration” is generally understood to denote the directional movement of H* molecules generated at metal active sites toward the support surface. This phenomenon is fundamentally driven by the disparity in H* adsorption strength, with H* molecules migrating from a primary active component with a strong binding affinity to an adjacent phase that exhibits a lower migration barrier.

**Fig. 1:**
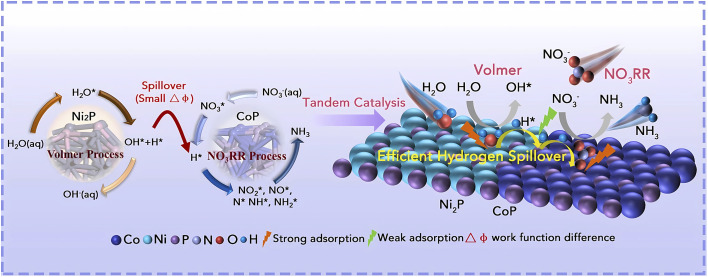
Schematic illustration of the hydrogen-spillover-mediated mechanism for selective NH_3_ production.^52^ Copyright 2025, *Nano Energy*.

As demonstrated in Fig. 1, the fundamental principle is the functional decoupling of the water dissociation site from the nitrate reduction site.^50,51^ Donor sites activate water and generate H*. Reduction sites adsorb NO_3_^−^ and convert NO_*x*_ intermediates. The interfacial migration channel enables directional H* transport. This spatial functional separation disrupts the direct competition between the HER and NO_3_RR on a single site: H* that is generated at the donor site migrates rapidly to the NO_*x*_-rich reduction site. At this site, the hydrogen atom is preferentially consumed in hydrogenation rather than accumulating and undergoing H–H coupling at the donor surface. From a thermodynamic perspective, the essence of hydrogen spillover can be understood as the establishment of an H* chemical potential gradient. This gradient drives H* from the high-chemical-potential donor site to the low-chemical-potential reduction site. The strong H* consumption capacity of NO_*x*_ intermediates sustains this gradient. Consequently, the design logic for NO_3_RR catalysts has progressively shifted from optimizing the intrinsic activity of a single active site toward the cooperative construction of a “donor site–migration channel–reduction site” tripartite architecture.

## 3. Catalyst design strategies

The hydrogen spillover effect functions as a bridge, thereby linking water dissociation with nitrate reduction. The generation, migration, and consumption of H* directly address the central challenges of an insufficient proton supply and competing HER in NRA. In recent years, there has been a rapid development of hydrogen spillover paradigms, evolving from conventional reverse spillover and bulk-phase hydrogen storage to dual site cooperativity and single atom hydrogen pumps. This evolution has enriched the toolkit of catalyst modification strategies and markedly improved reaction kinetics and ammonia selectivity. Presently, the majority of hydrogen spillover catalytic systems depend on the structural engineering of precious metals and their supports. Spillover behavior demonstrates a high degree of sensitivity to catalyst architecture, physicochemical properties, the reaction environment, and external operating conditions. In consideration of this framework, the design philosophy for NRA catalysts has evolved from a focus on optimizing the intrinsic activity of a single active site to a more collaborative construction of a donor site–transport channel–reduction site tripartite architecture. This review accordingly examines the research progress on hydrogen spillover enhanced NRA across different regulatory dimensions, with an emphasis on catalyst design strategies that harness hydrogen spillover to achieve efficient nitrate-to-ammonia conversion.

### 3.1 Cu-based reduction domains and H* supply regulation

Among various electrocatalytic materials for nitrate reduction, Cu-based catalysts represent a typical class of reduction domains in NRA. Copper and its oxides possess moderate adsorption affinity for nitrate and NOx intermediates, which benefits nitrate activation and deoxygenation.^53–56^ Their main limitation lies in the inadequate generation, retention, and delivery of reactive H* required for deep hydrogenation under cathodic conditions. Support-assisted water activation, secondary phase coupling, electronic modulation, and local interfacial microenvironment engineering can increase local H* availability around Cu-based reduction sites and partly relieve this limitation.^57^ In this context, hydrogen spillover provides a mechanistic route to couple NO_*x*_ activation with regulated H* delivery at catalytic interfaces.^58^

**Fig. 2:**
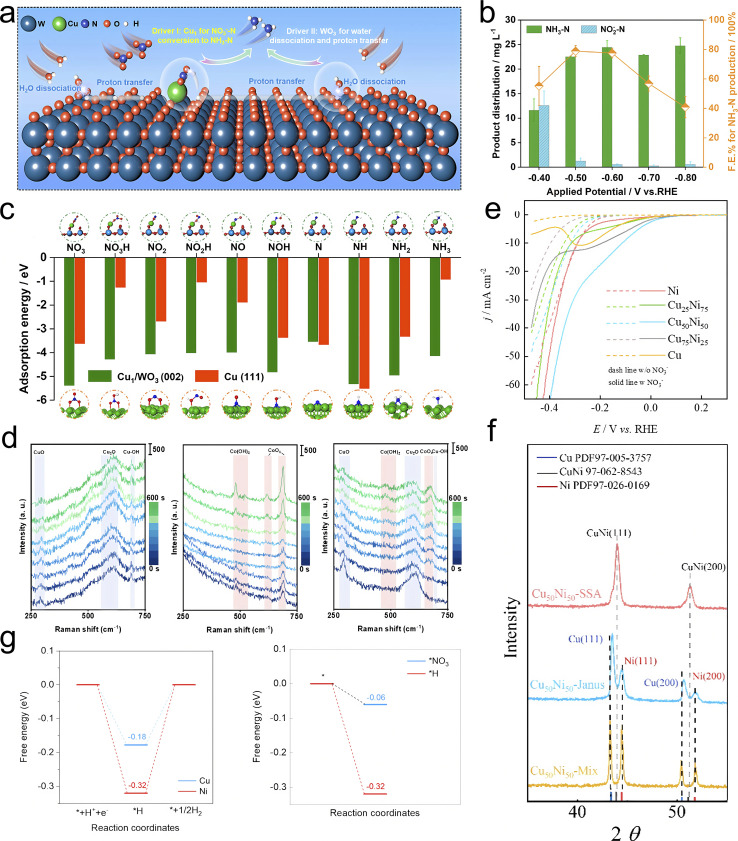
(a) Adsorption energies of *NO_3_ and nitrogen-containing intermediates on Cu (111) and Cu_1_/WO_3_ (002) surfaces. (b) Yield and FE for NH_3_ production under different applied potentials. (c) Schematic illustration of the dual-driven NRA mechanism on Cu_1_/WO_3_. Copyright 2025, *Angewandte Chemie*. (d) *In situ* Raman spectra collected during NRA at different reaction times (0–600 s). Copyright 2025, *Green Chem*. (e) LSV curves of Cu/C, Cu_75_Ni_25_–SSA/C, Cu_50_Ni_50_–SSA/C, Cu_25_Ni_75_–SSA/C, and Ni/C. (f) XRD patterns of Cu_50_Ni_50_–Mix/C, Cu_50_Ni_50_–Janus/C, and Cu_50_Ni_50_–SSA/C. (g) Calculated Gibbs free energies of *H and *NO_3_ adsorption on Cu and Ni sites. Copyright 2024, *ACS Catalysis*. (Panels a and b are adapted from the original theoretical study and are shown as schematic reaction pathway representations. The molecular drawings are not intended to define the exact molecular geometry).

The primary design logic for Cu-based catalysts involves breaking competitive adsorption on a single active site and achieving spatial decoupling of substrate activation from H* supply. The WO_3_–Cu_1_ single atom catalyst, as constructed by Hou *et al.*,^59^ serves as a prime example of the hydrogen supply compensation strategy. Here, the Cu_1_/WO_3_ configuration is considered as an idealized model for discussing support-assisted H supply near Cu sites, rather than as a direct structural assignment of the catalyst under NRA conditions. WO_3_ does not merely serve as an inert support; it promotes water dissociation and modulates the local proton supply, furnishing a utilizable hydrogen source for electron-deficient Cu_1_ sites. Within this idealized model, the Cu_1_ site was proposed to adsorb NO_3_^−^ and promote deep reduction, whereas WO_3_ was proposed to assist hydrogen supply and proton regulation (Fig. 2a). This catalyst achieves an NH_3_ yield of 1274.4 mg h^−1^ g_Cu_3__^−1^ and an NH_3_ selectivity of 99.2%, surpassing the performance of most reported catalysts (Fig. 2b). As demonstrated in Fig. 2c, this high performance is attributed to the continuous H* supply around Cu sites, which facilitates the surmounting of deep hydrogenation barriers by 
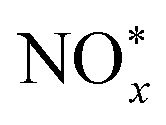
 intermediates.

The Cu–Co bimetallic catalyst reported by Li *et al.*^60^ demonstrates the dynamic regulation of hydrogen supply capacity through *in situ* phase transformation. In this system, Co undergoes *in situ* conversion to Co(OH)_2_ during the reaction, persistently promoting water dissociation and H* generation. This process compensates for the deficiencies of Cu sites in water activation and endows Cu sites with a sustained hydrogenation driving force. *In situ* Raman spectroscopy was utilized to elucidate the transformation process (Fig. 2d). Under reducing conditions, surface oxides on Cu are gradually reduced to metallic Cu. Metallic Cu exhibits a preferential adsorption of NO_3_^−^ and converts it to NO_2_^−^. Concurrently, metallic Co undergoes an oxidatively reconstructed process to Co(OH)_2_. This *in situ* generated Co(OH)_2_ phase continuously supplies H* to drive the subsequent deep hydrogenation steps. Concurrently, *in situ* Raman spectroscopy detected the characteristic peaks of key intermediates, including *NO_2_^−^ (1325 cm^−1^), *NO (1528 cm^−1^), and NH_2_ (1610 cm^−1^). The synergistic mechanism of dynamic interfacial reconstruction and hydrogen spillover proved to be beneficial to Cu_5_–Co_5_, which achieved a maximum FE of 94.1% and an NH_3_ yield of 30.9 mg h^−1^ cm^−2^. This robust performance was demonstrated in both simulated wastewater and a 20-hour stability test.

The decoupling of hydrogen supply and reduction is not solely determined by chemical compositional differences; it is also contingent on a rational spatial architecture that preserves the functional purity of each component. Sun *et al.*^61^ revealed this structural principle by comparing a homogeneous CuNi alloy with a Cu-rich/Ni-rich phase-separated structure. In the homogeneous alloy (Fig. 2e), the water dissociation function of Ni and the NO_3_^−^ activation function of Cu are mutually diluted by atomic level mixing. In contrast, Janus Cu@Ni retains nanoscale phase separation features with coexisting Cu-rich and Ni-rich domains (Fig. 2f). DFT calculations (Fig. 2g) demonstrate that Ni exhibits a stronger H* adsorption affinity of −0.32 eV compared to that of Cu (−0.18 eV). Additionally, Cu is more conducive to nitrate adsorption and activation. Consequently, H* is preferentially generated on the Ni-phase surface and spontaneously migrates *via* hydrogen spillover to the Cu–Ni interface, markedly reducing the energy barriers for N–O bond cleavage and subsequent hydrogenation. This cooperative enhancement of the tandem catalysts enables the Janus catalyst to achieve an NH_3_ production rate that is nearly five times that of the homogeneous alloy.

The aforementioned strategies are predicated on the introduction of a secondary metal or oxide as an external H* source. However, each additional interface inevitably introduces complications, including lattice mismatch, interfacial charge accumulation, and structural complexity. An alternative approach involves enhancing H* accessibility on Cu by tuning its intrinsic electronic structure, thereby circumventing the necessity for an external hydrogen supply phase.

**Fig. 3:**
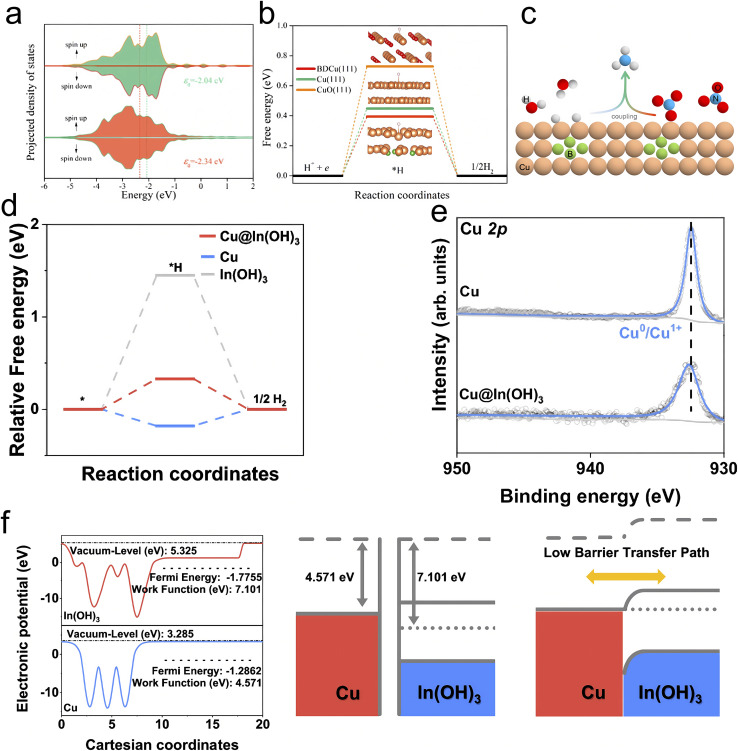
(a) Projected density of states (PDOS) of Cu (111) and BDCu (111). (b) Gibbs free energy diagrams for H* formation on Cu (111) and BDCu (111). (c) Proposed reaction mechanism for NH_3_ synthesis on the BDCu surface. Copyright 2025, *ACS Catalysis*. (d) Calculated WF of Cu and In(OH)_3_ and the corresponding energy band alignment diagram at the Cu/In(OH)_3_ interface. (e) Cu 2p XPS spectra of Cu and Cu@In(OH)_3_. (f) Gibbs free energy profiles of the HER on Cu, In(OH)_3_, and Cu@In(OH)_3_. Copyright 2025, *Nature Communications*.

Cu_2_O functions primarily as a pre-catalyst, with the actual active phase under working conditions being a Cu_2_O/Cu composite. At mild reduction potentials, the surface remains Cu^+^-rich and catalyzes the two-electron reduction of NO_3_^−^ to NO_2_^−^. As the potential shifts further in the negative direction, both the surface and the bulk are largely reduced to Cu^0^. Furthermore, both NH_3_ selectivity and yield exhibit a positive correlation with the Cu^0^ content. Lam *et al.*^62^ examined a Cu/Cu_2_O/PVDF architecture that adds a further layer of complexity: the electrode microenvironment. The Cu/Cu_2_O interface modulates Cu^0^/Cu^+^ cooperativity, electron transfer, and NO_*x*_ intermediate stability. In contrast, the PVDF membrane alters the interfacial water structure, local ion distribution, and reactant accessibility. This system demonstrates that the catalytic behavior of Cu-based catalysts is governed not only by metal valence or crystal phase but also by the interfacial water structure and local mass-transport environment at the electrode–electrolyte boundary. In the context of a multi-proton-coupled electron transfer reaction, such as NRA, microenvironmental regulation of this nature has the potential to influence the dynamics of H* generation, the duration of its surface residence, and the rate of its consumption. Consequently, this regulation can result in a shift in the equilibrium between NO_3_^−^ reduction and the HER. Boron doping presents an alternative approach: Zhang *et al.*^63^ synthesized a BDCu catalyst, in which electron transfer from Cu^0^ to B generates highly active Cu^*x*+^ species. PDOS analysis revealed that B doping shifts the Cu d-band center from −2.04 eV to −2.34 eV (Fig. 3a). This downward shift produces a dual effect: it weakens the Cu–H* bond, lowering the H* migration barrier on Cu (Fig. 3b), while simultaneously strengthening water activation and promoting H* generation (Fig. 3c). B doping effectively addresses the “slow H* generation” and “difficult H* migration” issues associated with Cu, while circumventing the necessity for an external hydrogen supply phase. This approach yields an FE of 96.58% and an NH_3_ yield of 25 741.5 µg h^−1^ mg_cat_^−1^. Yu *et al.*^64^ leveraged the substantial work function disparity between Cu and the n-type semiconductor In(OH)_3_ to engineer an ohmic–contact interface (Fig. 3d). Electrons spontaneously transfer from Cu to In(OH)_3_, forming a stable charge polarization region at the interface. This suppresses the self-reduction of Cu^*δ*+^ and enables the long-term coexistence of Cu^0^–Cu^*δ*+^ active sites. XPS characterization confirmed interfacial charge transfer and the stable retention of Cu^0^/Cu^*δ*+^ dual states (Fig. 3e). The presence of highly polarized Cu^0^–Cu sites enhances NO_2_ adsorption, while weakly polarized Cu^0^–Cu sites sustain continuous H* generation and supply. This collaborative effect leads to the efficient intermediate hydrogenation process, facilitated by hydrogen spillover (Fig. 3f). These Cu-based systems highlight a central requirement for spillover-assisted NRA. Nitrate activation and NO_*x*_ hydrogenation must be matched with a regulated supply of reactive H*. When this balance cannot be achieved on Cu-based sites alone, hydrogen-donating domains and nitrate reduction domains need to be spatially coupled through a suitable migration interface.

### 3.2 Donor phase–reduction phase coupling

The nitrate reduction system is organized around a “donor site–migration interface–reduction site” coupled architecture. Interfacial synergistic hydrogen spillover is the most intensively studied form of spillover in the field of NRA.^65^ The term specifically refers to the interfacial migration of H* between different components or active sites on the catalyst surface. In this subsection, phase coupling is used as a structural classification. The focus is on how H donor sites, migration interfaces, and nitrate reduction sites are integrated into a coupled architecture. Therefore, the key issues are phase composition, contact mode, interfacial continuity, and the stability of the coupled domains under NRA conditions. The coupling of the water dissociation site with the nitrate hydrogenation site overcomes the limitations of a single material in simultaneously achieving efficient hydrogen production and efficient hydrogenation.^66,67^ Constructing a tightly integrated interface *v*, such as a heterojunction or atomically bonded junction, lowers the energy barrier for hydrogen spillover between components, enabling efficient directional H* transport.

**Fig. 4:**
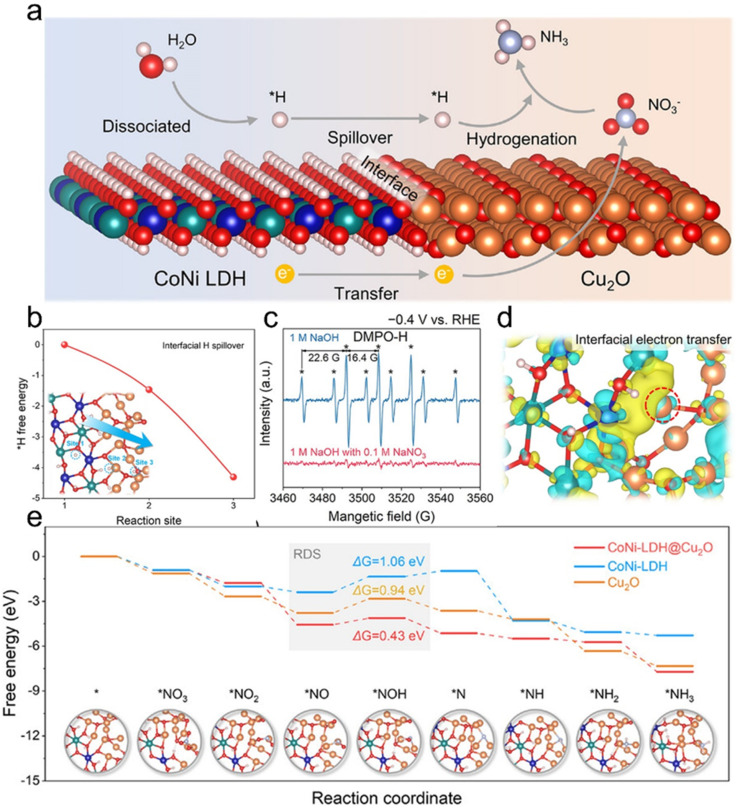
(a) Schematic of interfacial electron transfer and hydrogen spillover on CoNi-LDH@Cu_2_O for enhanced NO_3_RR. (b) Gibbs free energy of *H at interfacial sites; inset: spontaneous hydrogen spillover from CoNi-LDH to Cu_2_O. (c) *In situ* EPR spectra of CoNi-LDH@Cu_2_O at −0.4 V *vs.* RHE with and without NO_3_^−^. (d) Charge density difference of CoNi-LDH@Cu_2_O (yellow: charge accumulation; blue: charge depletion; isosurface: 0.004 e^−^ bohr^−3^). (e) Gibbs free energy diagrams of NRA on different catalysts; insets: optimized intermediate adsorption structures on CoNi-LDH@Cu_2_O. Copyright 2025, *Advanced Materials*.

**Fig. 5:**
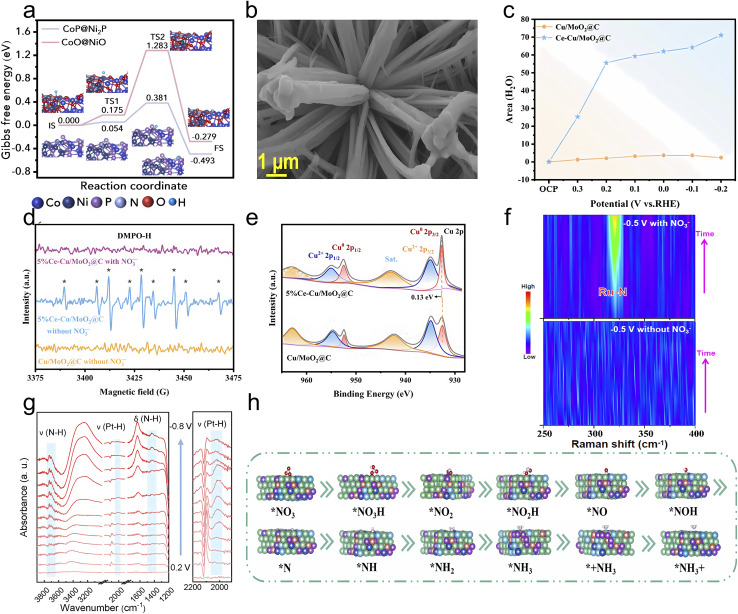
(a) Effect of work function difference (Δ*Φ*) on interfacial hydrogen spillover for CoO@NiO and CoP@Ni_2_P. Copyright 2025, *Nano Energy*. (b) Surface TEM image of Ru–CoFe LDH@Cu_*x*_O NW. Copyright 2025, *Chemical Engineering Journal*. (c) Integrated peak areas of adsorbed water on Cu/MoO_2_@C and 5% Ce–Cu/MoO_2_@C at different potentials. (d) Quasi *in situ* EPR spectra of Cu/MoO_2_@C and 5% Ce–Cu/MoO_2_@C under different conditions. (e) Cu 2p XPS spectra of Cu/MoO_2_@C and 5% Ce–Cu/MoO_2_@C. Copyright 2025, *Advanced Functional Materials*. (f) Time-dependent *operando* Raman contour maps of RuO_*x*_/Pd at −0.5 V with and without NO_2_^−^. Copyright 2023, ACS Nano. (g) *In situ* FTIR spectra of NRA on Pt/np-Co_2_P at different applied potentials. Copyright 2025, *Advanced Materials*. (h) Optimized adsorption structures of NRA intermediates on MA. Copyright 2025, *Journal of the American Chemical Society*.

The geometry of the interface directly governs the effective contact area between the donor and reduction phases. Lv *et al.*^68^ cultivated CoNi layered double hydroxide (CoNi-LDH) nanosheets *in situ* on a Cu_2_O nanowire array, thereby engineering a self-supporting heterojunction electrode (CoNi-LDH@Cu_2_O) with a tunable interface (Fig. 4a). In the proton transfer channel, the incorporation of CoNi-LDH led to a significant reduction in the water dissociation barrier. This resulted in the spontaneous spillover of H* to the Cu_2_O side, driven by the interfacial H* free-energy gradient (Fig. 4b). *In situ* electron paramagnetic resonance (EPR) experiments detected a clear DMPO-H signal in the absence of NO_3_^−^, which nearly vanished upon NO_3_^−^ addition. This finding directly confirms that H* is continuously consumed by NO_*x*_ intermediates (Fig. 4c). DFT calculations demonstrated that this spontaneous hydrogen spillover process reduced the energy barrier of the RDS (*NO → *NOH) to 0.43 eV (Fig. 4d). In the electron transfer channel, the substantial work function disparity between CoNi-LDH and Cu_2_O promoted directional electron transfer to unsaturated Cu sites at the Cu_2_O interface (Fig. 4e). The synergistic superposition of these two interfacial channels resulted in an NH_3_ FE of 97.8% and an NH_3_ yield of 75.2 mg h^−1^ cm^−2^ at −0.3 V *vs.* RHE, with stable operation at a current density approaching 1 A cm^−2^. It is imperative to note that a substantial work function difference does not invariably prove advantageous. An excessively pronounced Δ*Φ* engenders a formidable Schottky barrier, which effectively captures H* at the interface. In a related study, Wang *et al.*^52^ subjected a CoO@NiO precursor to phosphidation, achieving a precise compression of the interfacial Δ*Φ* from 0.35 eV to 0.25 eV. This significant reduction in the interfacial Δ*Φ* resulted in a substantial decrease in the cross-interface H* migration barrier, lowering it from 1.283 eV to 0.381 eV (Fig. 5a). The findings of these two studies indicate an optimal window for the interfacial work function difference, sufficient to facilitate spontaneous H* migration, yet not so extensive as to impede the formation of a charge accumulation barrier.

Core–shell and confined architectures leverage the spatial confinement effect of three-dimensional nanocavities to enhance spillover efficiency.^69^ Bai *et al.*^70^ fabricated a hedgehog-like Ru–CoFe LDH@Cu_*x*_O core–shell nanoneedle array catalyst (Fig. 5b). The Ru-doped CoFe LDH shell functions as the hydrogen donor site, exhibiting a water dissociation barrier of only −0.3072 eV. In contrast, the Cu_*x*_O core serves as the reduction site. The LDH shell surface H–H coupling barrier is measured to be 1.138 eV, thereby thermodynamically blocking the HER and ensuring that H* preferentially migrates *via* reverse hydrogen spillover to the Cu_*x*_O core. This catalyst demonstrated an FE of 98.95% and an NH_3_ yield of 6.59 mmol h^−1^ cm^−2^ at −0.5 V *vs.* RHE, exhibiting stability over an 80-hour period.

Beyond the construction of complex heterointerfaces, promoter modification offers a more operationally straightforward means of regulating hydrogen spillover. Rare-earth elements and metal oxides can modulate the electronic structure of catalysts and modify the surface adsorption environment, thereby tuning the kinetics of H* generation and consumption. Song *et al.*^71^ introduced Ce as isolated atoms into a Cu/MoO_2_@C system, constructing a Ce–Cu dual-site tandem catalyst. The Ce sites function not only as electronic modulators but also enhance water adsorption and dissociation, thereby continuously supplying H* to adjacent Cu sites. As illustrated in Fig. 5c, the water adsorption peak intensity of the 5% Ce-doped sample is significantly higher than that of the undoped sample. *In situ* EPR experiments further confirmed that H* generated at Ce sites rapidly spills over to Cu sites to participate in NO_*x*_ hydrogenation (Fig. 5d). As demonstrated in Fig. 5e, the introduction of Ce results in a 0.13 eV increase in the Cu 2p_3/2_ binding energy, indicating a robust electronic interaction between Ce and Cu. This interaction optimizes H* coverage and migration capability, thereby achieving dynamic alignment between active hydrogen supply and NO_*x*_ intermediate conversion rate. The optimized 5% Ce–Cu/MoO_2_@C delivered an NH_3_ yield of 20.3 ± 0.7 mg h^−1^ mg_cat_^−1^ and an FE of 92 ± 3% at −0.4 V *vs.* RHE.

Static structural characterization is often inadequate for accurately capturing the active interface under NRA operating conditions. A significant number of metal or oxide catalysts are known to undergo hydroxylation, redox transformations, or surface phase reconstruction under electroreduction conditions. This process results in the generation of new donor or reduction phases *in situ*. In the context of donor-reduction coupled systems, it is important to note that the structure designed prior to the reaction merely constitutes the initial state. The interface that genuinely participates in H* generation and migration has the potential to form *in situ* under *operando* conditions. As Li *et al.*^62^ have previously reported and as was discussed above, the Cu–Co bimetallic nanocomposite catalyst undergoes surface reconstruction during the reaction, with metallic Co converting *in situ* to Co(OH)_2_. *In situ* Raman results revealed that Cu sites preferentially adsorb nitrate and reduce it to nitrite. The *in situ*-generated hydroxylated cobalt phase, possessing excellent water dissociation capability, continuously supplies H* for deep nitrite reduction. The findings of these studies suggest that the true active interface in NRA frequently originates from reaction-induced phase transformations, hydroxylation, and redox reconstruction. Furthermore, the results indicate that dynamically evolving interfaces play a non-negligible regulatory role of notable significance in hydrogen spillover coupled systems.

Noble metals such as Pt, Pd, and Ru possess excellent water activation and H* generation capabilities but are also prototypical HER active sites. The optimization focus is therefore on achieving directional H* transport through interfacial coupling to suppress hydrogen-atom recombination. Chu *et al.*^72^ designed a RuO_*x*_/Pd catalyst in which sub nanometric RuO_*x*_ clusters are anchored on Pd metallene. The process of water dissociation and H* generation is facilitated by Pd, while RuO_*x*_ is responsible for NO_3_^−^ adsorption. *In situ* Raman spectroscopy (Fig. 5f) detected a Ru–N vibrational band near 320 cm^−1^ but no Pd–N signal. This finding confirms that NO_*x*_ intermediates are preferentially hydrogenated at RuO_*x*_ sites by interface accumulated H*, reducing the *NO → *NHO barrier to 0.33 eV. This catalyst demonstrated an NH_3_ FE of 98.6% and an NH_3_ yield of 23.5 mg h^−1^ cm^−2^ at −0.5 V *vs.* RHE. Li *et al.*^73^ further quantified the cross-interface hydrogen transfer in Pt/np-Co_2_P, revealing that the H* migration barrier from Pt to adjacent Co sites is merely 0.11 eV. They also found that the barrier for spillover-H-mediated NO hydrogenation (0.29 eV) is far lower than the direct hydrogenation pathway at Co sites (0.91 eV). *In situ* Fourier transform infrared spectroscopy (FTIR) at 2018 cm^−1^ directly traced the complete Pt–H migration pathway (Fig. 5g). This system successfully maintained NH_3_ FE above 90% across a 600 mV wide potential window and achieved near 100% selectivity at approximately 1 A cm^−2^. Wang *et al.*^52^ further demonstrated that noble-metal phosphide interfaces and open pore structures can synergistically sustain H* flux, delivering an NH_3_ yield of 0.908 mmol h^−1^ mg_cat_^−1^ at −0.645 V *vs.* RHE. This finding was made using Ni_2_P/Pd_6_P porous nanorods.

As catalytic systems have evolved to multicomponent catalysts, donor–reduction coupling may manifest as statistical cooperativity among multiple sites. Liu *et al.*^74^ reported Ni-WS_2_, in which metal–support interactions induce interfacial charge redistribution, achieving an NH_3_ yield of 32 mg h^−1^ cm^−2^ in a membrane electrode assembly with stable operation exceeding 100 h.^75^ Lu *et al.*^76^ developed an MA-TiO_2_ multicomponent alloy system that achieved an NH_3_ FE exceeding 93% under acidic, neutral, and alkaline conditions with 100 h stability. The presence of nanoporous surfaces in high-entropy alloys has been demonstrated to facilitate rapid H* migration within a multi-site network (Fig. 5h). This phenomenon has been shown to enhance the adaptability of NRA to fluctuations in potential, pH, and local concentration.

### 3.3 Migration direction regulation

After the donor and reduction phases have been spatially coupled, the next mechanistic requirement is to regulate the direction of H migration. The classification criterion in this subsection is therefore not the presence of a heterointerface itself, but the energetic or electronic asymmetry that drives H toward NO_*x*_ hydrogenation sites. Subsequent to the spatial coupling of the donor and reduction phases, the subsequent central question pertains to the steering of H* migration along a direction conducive to NO_*x*_ hydrogenation, as opposed to permitting random diffusion or consumption by the HER. The phenomenon of effective directional hydrogen spillover is contingent upon the presence of two distinct conditions. Firstly, a discernible electronic-structure difference must exist across the interface, thereby providing the thermodynamic driving force. Secondly, a migration channel with a low barrier must be present between the donor and acceptor sites. The work function difference, Fermi level offset, d-band center disparity, built-in electric field (BEF), adsorption energy gradient, and bridging-atom architecture have all been demonstrated to play a role in regulating the direction and kinetics of H* migration.^77^

**Fig. 6:**
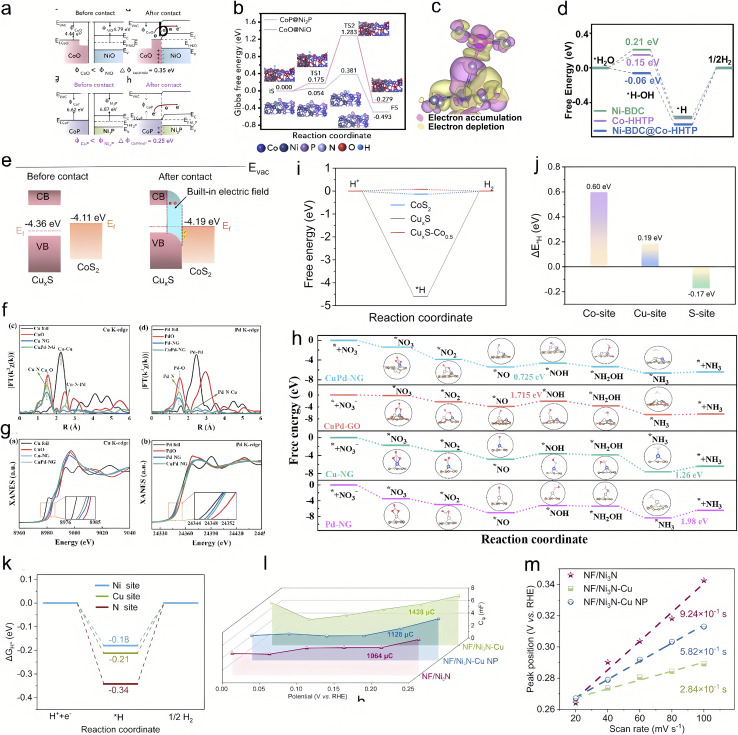
(a) Energy band diagrams of CoO/NiO and CoP/Ni_2_P before and after contact, illustrating the effect of work function difference (Δ*Φ*) on interfacial hydrogen spillover. (b) Gibbs free energy diagrams of hydrogen spillover from Ni to Co sites on CoP@Ni_2_P and CoO@NiO. Copyright 2025, *Nano Energy*. (c) Calculated charge density difference of Ni-BDC@Co-HHTP (purple: electron accumulation; yellow: electron depletion). (d) Gibbs free energy diagrams of water dissociation on Ni-BDC, Co-HHTP, and Ni-BDC@Co-HHTP. Copyright 2024, *Angewandte Chemie International Edition*. (e) Energy band diagrams of Cu_*x*_S and CoS_2_ before and after contact with built-in electric field formation. Copyright 2024, *ACS Catalysis*. (f) EXAFS spectra at the Cu K-edge and Pd K-edge for the catalysts and reference samples. (g) XANES spectra at the Cu K-edge and Pd K-edge. (h) Gibbs free energy diagrams of NRA pathways on CuPd-NG, CuPd-GO, Cu-NG, and Pd-NG. Copyright 2025, *ACS Catalysis*. (i) HER free energy diagrams on CoS_2_, Cu_*x*_S, and Cu_*x*_S–Co_0.5_. Copyright 2024, ACS Catalysis. (j) *H adsorption energy at Co, Cu, and S sites of Cu_*x*_S–Co_0.5_. Copyright 2026, *Angewandte Chemie International Edition*. (k) Hydrogen adsorption Gibbs free energy diagrams of Ni_3_N–Cu at Ni, Cu, and N sites. (l) *Q*_H_ and double-layer capacitance (*C*_dl_) of NF/Ni_3_N–Cu, NF/Ni_3_N–Cu NP, and NF/Ni_3_N at different potentials. (m) CV peak position *vs.* scan rate plots for NF/Ni_3_N–Cu, NF/Ni_3_N–Cu NP, and NF/Ni_3_N. Copyright 2025, *Angewandte Chemie International Edition*.

In hybrid structures, electron redistribution is often triggered by contact between dissimilar components owing to differences in work function.^78,79^ In a seminal study, Wang *et al.*^52^ pioneered the development of a single-phase bilayer CoP@Ni_2_P/NF nanoarray. This innovative design entails the concerted action of Ni sites, which are instrumental in H_2_O dissociation and H* enrichment, and Co sites, which facilitate NO_3_^−^ adsorption and H* reception. Ultraviolet photoelectron spectroscopy (UPS) measurements revealed that the work function difference of phosphidized CoP@Ni_2_P/NF (Δ*Φ* = 0.25 eV) is markedly smaller than that of CoO@NiO/NF (Δ*Φ* = 0.35 eV) (Fig. 6a). The reduced Δ*Φ* induces moderate interfacial charge reconstruction, thereby lowering the cross-interface H* migration barrier from 1.283 eV to 0.381 eV (Fig. 6b). This catalyst demonstrated a half-cell ammonia synthesis energy efficiency of 40.66% and an NH_3_ yield of 172.64 mg h^−1^ cm^−2^. Fermi level differences can similarly construct interfacial driving forces. Liu *et al.*^80^ selected two metal organic frameworks (MOFs) with different Fermi levels and grew Co-HHTP nanorods on Ni-BDC nanosheets to construct a MOF-on-MOF heterostructure. As illustrated in Fig. 6c, charge density difference maps revealed that Fermi-level-difference-induced charge redistribution occurred. Specifically, electron-rich Co sites exhibited a propensity for H_2_O dissociation, thereby generating H*, while electron-deficient Ni sites demonstrated a higher affinity for NO_3_^−^ adsorption and NO_*x*_ hydrogenation. In the context of the BEF, H* migrates from Co sites to Ni sites, resulting in a substantial reduction in the *NO_2_ → *HNO_2_ hydrogenation barrier (Fig. 6d). In a related study, Wang *et al.*^81^ prepared copper–cobalt binary sulfide nanosheets that were rich in Cu_7.2_S_4_/CoS_2_ heterointerfaces. The UPS-derived band structures (Fig. 6e) demonstrated that electrons flow from CoS_2_ (Fermi level −4.11 eV) to Cu_*x*_S (−4.36 eV), thereby forming a Schottky junction and generating a localized nanoscale BEF. This BEF tunes H adsorption strength to a suitable range, enabling H* to migrate preferentially to NO_3_^−^ activation sites rather than undergoing coupling to evolve H_2_ (Fig. 6i).

Electronic-structure differences provide the driving force, but migration still requires concrete structural pathways. The bridging of atoms, such as N and S, can shorten the distance between donor and reduction sites. This, in turn, can modulate the H* migration barrier through local charge polarization. Zang *et al.*^82^ synthesized a CuPd-NG catalyst in which a Pd–N–Cu motif connects the donor Pd site and the reduction Cu site. Extended X-ray absorption fine structure (EXAFS) analysis confirmed the atomistic dispersion of both Pd and Cu are atomically dispersed (Fig. 6f). Cu K-edge X-ray absorption near-edge structure (XANES) spectra demonstrated the presence of a weak BEF formed by the N bridge (Fig. 6g), thereby promoting H_2_O dissociation at Pd sites and enabling rapid directional H* migration through the Pd–N–Cu bond (Fig. 6h).

Zhou *et al.*^83^ engineered asymmetric active centers within a Cu–Co_3_S_4_ system through a process known as sulfur-bridge engineering. The Co_3_S_4_ framework functions as the hydrogen-donor component, while isolated Cu atoms (1.01 wt%) act as NO_*x*_ conversion centers. DFT calculations revealed that the S bridge forms a descending thermodynamic gradient for H* migration (Fig. 6j). The catalyst was operated continuously for 300 hours at a current density of −200 mA cm^−2^, thereby retaining an NH_3_ FE of 95.18%. Xiong *et al.*^84^ demonstrated the relay-station design principle in an NF/Ni_3_N–Cu system. The Ni–N–Cu interfacial bridging bonds facilitate the formation of short-range directional channels, with N sites functioning as natural relay stations. The H* adsorption free energy on Cu sites (Δ*G*_H*_ = −0.34 eV) is positioned precisely between that of Ni sites (−0.18 eV) and N sites (−0.34 eV). This positioning enables stepwise directional H* transport along the Δ*G*_H*_ gradient (Fig. 6k). Electrochemical impedance spectroscopy (EIS) was utilized to quantitatively extract the hydrogen adsorption charge QH. This analysis revealed a significant discrepancy in the amount of charge between two distinct configurations: tightly anchored NF/Ni_3_N–Cu (1428 µC) and physically attached NF/Ni_3_N–Cu NP (1128 µC). The observed discrepancy in QH, amounting to 26.6%, was attributed entirely to the efficiency of interfacial coupling (Fig. 6l). Variable-scan-rate cyclic voltammetry (CV) further revealed that the slope of the H desorption peak potential *versus* log *v* for Ni_3_N–Cu is substantially lower than that for pristine Ni_3_N, confirming that H spilled over to the Cu surface exhibits faster desorption kinetics (Fig. 6m). This catalyst achieved an NH_3_ FE of 98.7% and an NH_3_ yield of 1.19 mmol h^−1^ cm^−2^ at −0.3 V *vs.* RHE.

In summary, the regulation of hydrogen spillover direction can be advanced on two fronts. At the thermodynamic level, the spontaneous driving force is provided by H* adsorption energy gradients established through work function differences, Fermi level offsets, and BEFs. At the structural level, bridging atoms such as N and S construct low-barrier structured migration channels. This transformation of cross-site H* transport from random surface diffusion into precisely directed conduction along chemical-bond pathways is a key feature of the system.

### 3.4 Shortening the migration pathway

The phenomenon of hydrogen spillover has the potential to supply additional H* for NRA. However, the efficiency of this process is contingent on two factors. Firstly, the generation of H* is essential, but it is equally crucial that it can reach NO_*x*_ intermediates within a suitable timescale. In the event that the migration distance is excessively extensive or the migration barrier is excessively elevated, H* is unable to participate in the sequential hydrogenation of *NO_2_, NO, and NHO in a timely manner. More critically, H* lingering on the surface is prone to undergoing H–H coupling or the Heyrovsky step, diverting into the HER pathway.^85–87^ Consequently, the reduction of the effective H* migration pathway emerges as a pivotal strategy to enhance NO_3_RR selectivity.

**Fig. 7:**
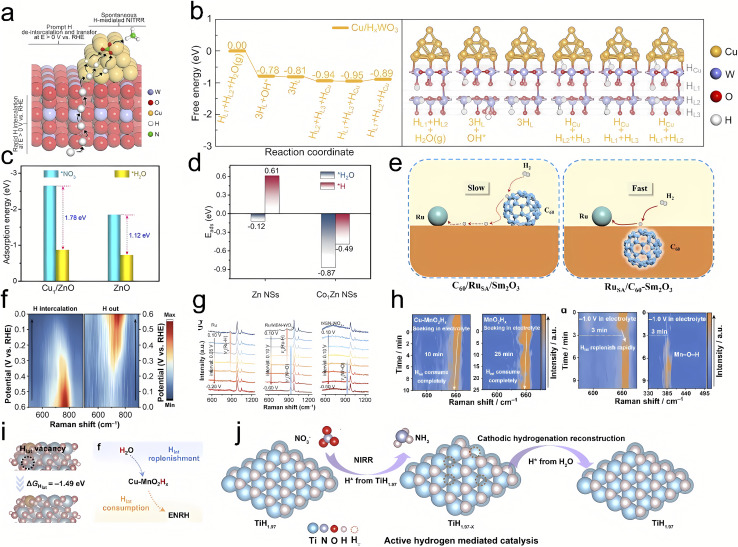
(a) Schematic of spontaneous H-mediated NRA on Cu/H_2_WO_3_ with prompt H intercalation and transfer. (b) Free energy diagram of H intercalation and spillover pathways on Cu/H_2_WO_3_; insets: optimized structures of key intermediates. Copyright 2024, *Angewandte Chemie*. (c) Adsorption energies of *NO_3_ and *H_2_O on Cu_2_/ZnO and ZnO. Copyright 2025, *Journal of the American Chemical Society*. (d) Calculated *E*_p_ of *H_2_O and *H on Zn NSs and Co_2_Zn NSs. Copyright 2025, *Angewandte Chemie International Edition*. (e) Schematic comparison of H_2_ evolution and hydrogen spillover rates on C_60_/Ru_SA/Sm_2_O_3_ and Ru___SA/C_60_-Sm_2_O_3_. Copyright 2026, *Applied Catalysis B: Environment and Energy*. (f) *Operando* Raman contour maps of H intercalation and extraction on Cu/H_2_WO_3_ at different potentials. (g) *In situ* Raman spectra of Ru/MSN-WO_3_ and MSN-WO_3_ at different applied potentials. Copyright 2026, *Nature Communications*. (h) Time-dependent *in situ* Raman spectra of Cu–MnO_2_H_*X*_ and MnO_2_H_*X*_ at −1.0 V in electrolyte, showing H_ad_ consumption; inset: Mn–O–H region. (i) Schematic of H_ad_ vacancy formation and replenishment cycle on Cu–MnO_2_H_*x*_ during NRA. Copyright 2025, *Journal of the American Chemical Society*. (j) Schematic of active hydrogen-mediated catalysis on TiH_1.97_ during NRA, illustrating cathodic hydrogenation reconstruction from TiH_1.97_ to TiH_1.97−x_ and back. Copyright 2024, *Nature Communications*.

Li *et al.*^88^ employed H_*x*_WO_3_ as a reversible hydrogen storage/release medium to construct a Cu/H_*x*_WO_3_ composite catalyst. As demonstrated in Fig. 7a, H* rapidly intercalates into the H_*x*_WO_3_ lattice and subsequently undergoes immediate deintercalation to migrate to the Cu surface. Free-energy calculations revealed that hydrogen produced by water dissociation intercalates without a barrier, and subsequent deintercalation requires a remarkably low barrier of only 0.06 eV (Fig. 7b). This lattice-hydrogen deintercalation mechanism yields an FE approaching 100% at 0.10 V *vs.* RHE.

Lattice-hydrogen relay shortens the effective H* migration path; however, an even more direct strategy involves compressing donor and reduction sites to the atomic scale. In the Cu_1_/ZnO system,^89^ isolated Cu sites are primarily responsible for NO_3_^−^ adsorption and NO_*x*_ conversion, while the ZnO matrix promotes water dissociation. The anchoring of Cu single atoms on the ZnO surface results in the proximity of the hydrogen-supply environment and the reduction center at the atomic scale. Cu_1_/ZnO demonstrates a more negative adsorption energy for *NO_3_ with a substantial difference relative to H_2_O (Fig. 7c), indicating that single-atom Cu sites exhibit preferential adsorption of nitrate. This system has been demonstrated to maintain elevated NH_3_ FE across a broad spectrum of neutral and wide potential windows.

In single-atom alloy systems, isolated atoms can function as local water dissociation centers, thereby facilitating H* migration through the adsorption energy difference with adjacent host sites.^90^ Zeng *et al.*^91^ developed a Co_1_Zn single-atom alloy nanosheet in which isolated Co atoms function as hydrogen pumps. This phenomenon is attributed to the significant H* adsorption energy difference between Co and adjacent Zn sites (Fig. 7d). The migration of H* from Co sites to Zn sites is spontaneous, resulting in a current density that exceeds 2.4 A cm^−2^. This process leads to an NH_3_ yield of 204.5 mg h^−1^ cm^−2^ and an FE of 98.7%.

Jiang *et al.*^92^ demonstrated the RuSA/Ni_3_B system. Individual atoms exhibit remarkable efficacy in the generation of H*, while the Ni_3_B substrate possesses substantial NO_*x*_ activation capability (Fig. 7e). The Ru–B/Ni coordination environment provides a short-range channel for H* migration. A comparative analysis of RuSA/Fe_2_B and RuSA/Co_3_B with RuSA/Ni_3_B reveals that the latter exhibits a lower H* migration barrier. This observation suggests that the effectiveness of single-atom donor centers is contingent on their electronic-structure compatibility with the hydrogen-accepting substrate.

Bulk phase hydrogen-storage materials can temporarily store a fraction of active hydrogen in oxygen vacancies, lattice interstices, or reversible hydrogen storage phases. Liang *et al.*^93^ investigated MSN-WO_3−*x*_, in which abundant surface oxygen vacancies serve as Lewis acid sites that promote water dissociation. *In situ* Raman spectroscopy (Fig. 7f) demonstrated that the W=O stretching peak at 778 cm^−1^ experiences a gradual weakening with cathodic potential due to proton intercalation. Furthermore, it exhibits reversible behavior upon potential reversal. *In situ* surface-enhanced Raman spectroscopy (SERS) under NRA conditions (Fig. 7g) demonstrated that Ru active sites effectively accelerate the deprotonation of the hydrogenated tungsten trioxide support. In their seminal work, Yu *et al.*^94^ pioneered the design of Cu-doped MnO_2_: Cu doping induces Jahn–Teller distortion, thereby enabling protons to intercalate into the MnO_2_ lattice and generate abundant lattice hydrogen. This hydrogen can be rapidly replenished through water dissociation. *In situ* Raman spectroscopy acquired at −1.0 V *vs.* RHE revealed a blueshift recovery of the V_2_ peak and reappearance of the Mn–O–H bond signal after lattice-hydrogen depletion, characterizing the lattice-hydrogen regeneration process (Fig. 7h). The adsorption free energy of lattice hydrogen reaches as low as −1.49 eV after vacancy formation (Fig. 7i), enabling rapid regeneration. Zhou *et al.*^95^ prepared a TiH_2_ electrocatalyst. Its lattice hydrogen can be converted to H* for NRA hydrogenation and can equilibrate reversibly with H* generated from water dissociation (Fig. 7j). This process enables dynamic hydrogen source replenishment.

The central objective of shortening the H* migration pathway is to deliver H* to the vicinity of NO_*x*_ intermediates as quickly as possible after its generation. It is imperative to note that the acceleration of deep hydrogenation, the effective suppression of HER competition, and the maximization of catalytic performance are only attainable when H* generation, temporary storage, migration, and consumption are continuously coupled at the atomic or nanometre scale.

### 3.5 Interfacial microenvironment regulation

In practical NRA, H* generation, migration, and consumption are not governed only by the static catalyst structure. They are also affected by interfacial water networks, hydrated cations, defects, grain boundaries, *operando* reconstruction, and multicomponent cooperative networks.^96–99^

**Fig. 8:**
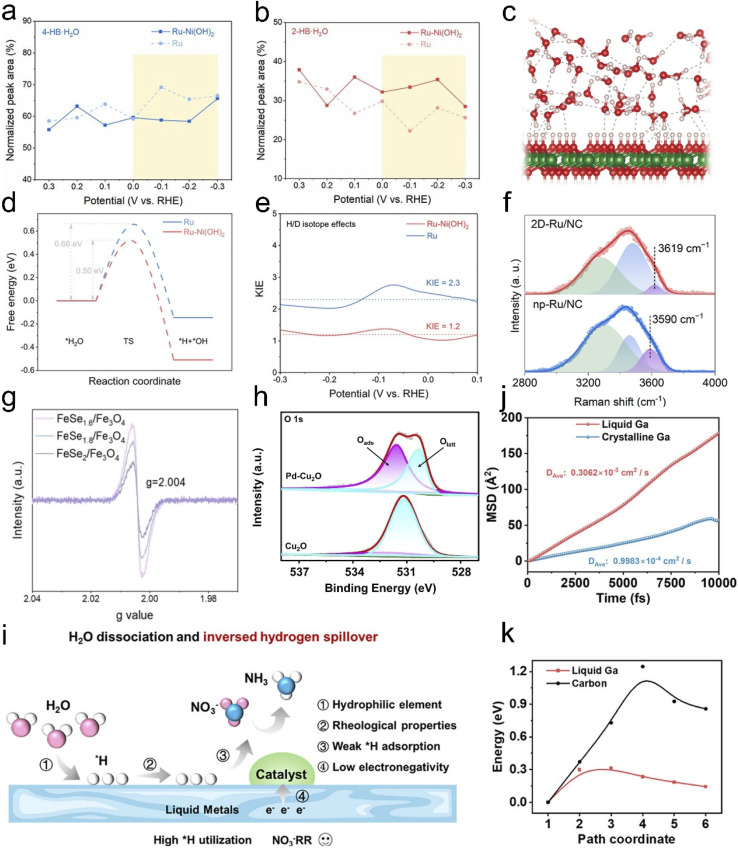
(a) Normalized peak areas of 4-HB·H_2_O and (b) 2-HB·H_2_O on Ru–Ni(OH)_2_ and Ru surfaces at different potentials. (c) Representative snapshots of interfacial H_2_O structures on the Ni(OH)_2_ surface. (d) H_2_O dissociation free energy barriers on Ru and Ru–Ni(OH)_2_ (0.50 eV *vs.* 0.66 eV). (e) Kinetic isotope effect (KIE) of Ru–Ni(OH)_2_ and Ru at different potentials. Copyright 2025, *Advanced Materials*. (f) *In situ* Raman spectra of interfacial water on 2D-Ru/NC and np-Ru/NC at v1.4 V *vs.* RHE. Copyright 2024, *Nature Communications*. (g) EPR spectra of FeSe_1.6_/Fe_3_O_4_, FeSe_1.8_/Fe_3_O_4_, and FeSe_2_/Fe_3_O_4_. Copyright 2024, *Chemical Society Reviews*. (h) O 1s XPS spectra of Pd–Cu_2_O and Cu_2_O. Copyright 2022, *Applied Catalysis B: Environmental*. (i) Schematic of H_2_O dissociation and inverse hydrogen spillover on liquid metal catalysts for NRA. (j) AIMD mean square displacement (MSD) of H* on liquid Ga and crystalline Ga. (k) H* migration energy barriers on liquid Ga and carbon. Copyright 2025, *Angewandte Chemie International Edition*.

In neutral or alkaline environments, water is the predominant source of protons. The regulation of the hydrogen-bond network, which involves the disruption of excessive interfacial water connectivity and the reduction of the distance between water molecules and the catalytic surface, has been identified as a promising approach to enhance H* generation. As demonstrated by Lv *et al.*,^100^ this phenomenon is not exclusive to the Ru–Ni(OH)_2_ system. The –OH groups present on the Ni(OH)_2_ nanosheet surface establish robust hydrogen bonds with interfacial water molecules, thereby disrupting the rigid tetrahedrally coordinated hydrogen-bond network and increasing the proportion of water molecules that are weakly hydrogen-bonded. *In situ* Raman spectroscopy revealed a significantly higher 2-HB/4-HB ratio on Ru–Ni(OH)_2_ relative to the Ru control (Fig. 8a and b). Furthermore, molecular dynamics (MD) simulations revealed that water molecules exhibit enhanced distribution on the Ni(OH)_2_ surface, thereby reducing the water dissociation barrier (Fig. 8c). DFT calculations indicated that H* spillover from Ni(OH)_2_ to Ru sites is thermodynamically spontaneous (Fig. 8d). This catalyst demonstrated an NH_3_ FE approaching 100% at 0 V *vs.* RHE and a peak energy efficiency of 44.6% at +0.1 V *vs.* RHE. The kinetic isotope effect (KIE) value was 1.2. This value is lower than the approximately 2.5 value observed for Ru/C (Fig. 8e).

In the context of noble-metal catalysts, a critical factor in enhancing selectivity is the precise modulation of the interfacial water layer. This strategy ensures a sufficient hydrogen supply, while concurrently disrupting the continuous water network that is a prerequisite for the HER.^101–103^ Li *et al.*^104^ developed a two-dimensional Ru-based material on nitrogen-doped carbon (2D-Ru/NC), thereby constructing a rectifying contact structure that renders the Ru surface markedly electron-deficient. *In situ* Raman spectroscopy resolved three coexisting interfacial water forms (Fig. 8f). *Ab initio* MD (AIMD) simulations demonstrated that hydrated K^+^ can detach from the 2D-Ru/NC surface within 0.4 ps. This exclusion leads to the formation of a loosely adherent interfacial water layer, which in turn suppresses the HER while promoting nitrate conversion. This results in an NH_3_ yield of 55.4 mg cm^−2^ h^−1^ at −1.1 V *vs.* RHE with FE retained at 90−94% for a duration exceeding 120 hours.

Defect sites modify the electronic and geometric structure of active sites, thereby modulating the local hydrogen environment.^105–107^ Yao *et al.*^108^ provided experimental evidence using an FeSe_2_/Fe_3_O_4_ Se-vacancy system: Fe_3_O_4_ facilitates water dissociation and H* generation, while Se-vacancy-rich FeSe_2_ promotes NO_3_^−^ deoxygenation. EPR detection revealed a robust characteristic signal at *g* = 2.004, assigned to unpaired electrons trapped within Se vacancies (Fig. 8g). DFT calculations demonstrated that the adsorption energy difference between Se vacancy sites (weak H* adsorption) and Fe_3_O_4_ oxygen sites (strong H* adsorption) governs directional hydrogen spillover. Similarly, Wang *et al.*^109^ developed a Pd–Cu_2_O-CEO composite catalyst that exhibited oxygen vacancies. X-ray photoelectron spectroscopy (XPS) O 1s analysis revealed abundant adsorbed oxygen species at vacancy sites (Fig. 8h), and the catalyst achieved an NH_3_ selectivity of 95.31%. Grain boundaries (GBs), defined as continuous low-coordination interfacial defects, exhibit a behavior that is complementary to that of point defects. Fu *et al.*^37^ prepared GB-rich Ni nanoparticle catalysts (GB Ni NPs): the distinctive low-coordination structure of GB regions strongly retains H*, inducing directional H* migration to NO_*x*_ intermediates. This catalyst demonstrated an NH_3_ production rate of 15.49 mmol h^−1^ cm^−2^ and an NH_3_ FE of 93.0%.

Liquid-metal systems offer a more adaptive dynamic interface model. Hu *et al.*^110^ reported a Co@Ga liquid-metal system featuring a liquid Ga (core)-solid Co (shell)-liquid electrolyte (L–S–L) three-phase dynamic interface (Fig. 8i). DFT calculations revealed that the Volmer step barrier on liquid Ga (0.70 eV) is lower than that on metallic Co. However, the Heyrovsky barrier is higher, causing H* on Ga to spill over spontaneously to Co sites (Fig. 8j). AIMD simulations showed that the H* diffusion coefficient on liquid Ga (approximately 3 × 10^−4^ cm^2^ s^−1^) is nearly three times that on solid Ga (Fig. 8k). This catalyst demonstrated a Co-mass-normalized NH_3_ yield of 51 mol h^−1^ g^−1^ and an NH_3_ FE of 94.5%, at 1 A cm^−2^ within a membrane electrode assembly over a duration of 400 hours (Table 1).^111^

**Table 1:** The catalytic performances of NRA electrocatalysts with hydrogen spillover under different strategies

Catalyst	Electrolyte	NH_3_ yield	FE (%)	Potential (*vs.* RHE)	Ref.
Cu_1_/WO_3_	0.5 M KNO_3_ + 0.1 M Na_2_SO_4_	1274.4 mg_N_ h^−1^ g_Cu_^−1^	93.7	−0.6	59
Cu–Co/NF	1.0 M KOH + 0.1 M NaNO_3_	14.8 mg h^−1^ cm^−2^	94.1	−0.6	60
Cu_50_–Ni_50_-Janus/C	1.0 M KOH + 0.1 M KNO_3_	1127 mmol g_cat_^−1^ h^−1^	92.5	−0.2	61
BDCu	0.05 M Na_2_SO_4_ + 0.1 M KNO_3_	25 741.51 µg h^−1^ mg_cat_^−1^	96.58	−1.15	62
Cu@In(OH)_3_	1.0 M KOH + 1 M KNO_3_	4.28 mmol h^−1^ mg_cat_^−1^	97.35	−0.6	63
CoNi-LDH@Cu_2_O	1.0 M KOH + 0.1 M KNO_3_	22.5 mg cm^−2^ h^−1^	∼100	−0.3	68
CoP@Ni_2_P@Cu_*x*_ONW	1.0 M NaOH + 0.1 M NO_3_^−^	172.64 mg h^−1^ cm^−2^	93.95	−0.4	52
Ru–CoFe LDH	1 M KOH + 0.1 M NO_3_^−^	6.59 mmol cm^−2^ h^−1^	98.95	−0.5	70
5%Ce–Cu/MoO_2_@C	1 M KOH + 0.1 M KNO_3_	20 277.32 µg h^−1^·mg_cat_^−1^	92.7	−0.4	71
RuO_*X*_/Pd	1.0 M KOH + 0.1 M KNO_3_	23.5 mg h^−1^ cm^−2^	98.6	−0.5	72
Pt/np-Co_2_P	1 M KOH + 0.1 M NO_3_^−^	3.449 mmol h^−1^ cm^−2^	98.91	−0.4	73
Ni-WS_2_	1 M KOH + 0.1 M KNO_3_	23.3 mg h^−1^ cm^−2^	85.1	−0.7	74
MA-TiO_2_	0.5 M K_2_SO_4_, 0.05 M KNO_3_	18 946.3 µg h^−1^ mg^−1^	96.1	—	75
Ni-BDC@Co-HHTP	1 M KOH + 0.1 M NaNO_3_	11.46 mg h^−2^ cm^−2^	98.4	−0.7	78
Cu_*x*_S-Co_0.5_	1 M KOH + 0.1 M KNO3	5.36 mg h^−1^·cm^−2^	95.6	—	79
CuPd-NG	1 M KOH + 0.1 M KNO_3_	0.96 mmol h^−1^cm^−2^	97.2	−0.6	80
Cu–Co_3_S_4_	1 M KOH + 0.1 M KNO_3_	94.52 mg h^−1^ mg_cat_^−1^	95.18	−0.8	81
NF/Ni_3_N–Cu	1 M KOH + 0.1 M KNO_3_	1.19 mmol h^−1^ cm^−2^	98.7	−0.3	82
Cu/H_*x*_WO_3_@CC	1.0 M KNO_3_	3332.9 ± 34.1 mmol g_cat_^−1^ h^−1^	∼100	0.1	89
Cu_1_ZnO	1.0 M KOH + 0.1 M NO_3_^−^	58.4 mg h^−1^ cm^−2^	96.7	−0.7	90
Co_1_Zn NSs	1.0 M KOH + 0.2 M KNO_3_	204.5 mg h^−1^ cm^−2^	94.2	−0.9	91
Ru/MSN-WO_3−x_	0.25 M K_2_SO_4_ +0.5 M phosphate +0.1 M KNO_3_	12.38 mg cm^−1^ h^−1^	91.50	−0.6	92
Cu–MnO_2_H_*x*_	(0.5 M K_2_SO_4_ + 0.2 M KNO_3_ + 0.2 M aceton	396.6 mmol g_cat_^−1^ h^−1^	91.1	−1.0	93
TiH_1.97_	1 M KOH + 0.1 M KNO_3_	83.6 mg cm^−2^ h^−1^	99.1	−0.7	94
Ru–Ni(OH)_2_	1 M KOH + 0.1 M KNO_3_	—	99.7	0	103
2D-Ru-NC	1 M KNO_3_	74.8 mg cm^−2^ h^−1^	94	−1.4	104
Ni-NPs-1.6	1 M NaNO_3_ + 1 M NaOH	15.49 mmol h^−1^ cm^−2^	93.0	−0.93	37
Se defected FeSe_1.6_/Fe_3_O_4_	0.5 M Na_2_SO_4_ + 0.1 M NO_3_^−^	31.46 mgh^−1^ cm^−2^	∼100	−1.2	107
Pd–Cu_2_O CEO	0.5 M K2SO4 + 50 mg L^−1^ NO_3_–N	925.11 µg h^−1^ mg_cat._^−1^	96.56	−1.3	109
Co@Ga LMMSs	1.0 M NaOH + 1.0 M NaNO_3_	51.1 mol h^−1^ g_m_^−1^	94.5	−0.3	110

A comparative assessment of the five strategies is summarized in Table 2. The five strategies target distinct points along the active hydrogen pathway, including H* formation, interfacial migration, and consumption by NOx intermediates. Cu-based electronic regulation benefits from the low cost of Cu and its favorable affinity for nitrate, while its limited water dissociation ability usually requires an auxiliary H supplying component. Donor and reduction phase coupling creates a clearer division between water activation and nitrate hydrogenation, provided that interfacial stability and *operando* phase evolution are verified. Migration direction control offers a mechanistic basis for guiding H* transport through H binding gradients, work function differences, and interfacial charge redistribution. Pathway shortening limits the probability of H* loss to the HER and places stricter demands on nanoscale contact and interface continuity. Microenvironment regulation is particularly relevant under high current operation, where electrolyte composition, local pH, ion distribution, and reactor geometry can reshape both H* residence and NO_*x*_ coverage. The comparison table therefore defines the operating window, main advantage, and key uncertainty of each strategy.

**Table 2:** Comparative assessment of catalyst design strategies

Strategy	Main strength	Main risk	Scale up outlook
Cu-based regulation	Low cost and strong NO_3_ affinity	Often limited by water activation	Good if H supply is added
Donor and reduction phase coupling	Clear division of H supply and NO_*x*_ reduction	Interface reconstruction and leaching	Moderate to high
Pathway shortening	Limits H loss to HER	Requires nanoscale synthetic precision	Moderate
Microenvironment regulation	Relevant to high current density	Sensitive to electrolyte and reactor design	High if validated in flow cells

## 4. Evidence for hydrogen spillover in NRA

Hydrogen activation and hydrogen spillover are foundational processes in numerous hydrogen-related reactions. Verifying hydrogen spillover faces three inherent difficulties. H* exists at trace levels and has a short lifetime. Hydrogen donation and acceptance are closely coupled in space and time at the electrochemical interface. Catalysts may also undergo phase transformations under *operando* conditions. Consequently, a comprehensive methodology is imperative to rigorously verify the existence of spillover in NRA electrocatalysts. Hydrogen spillover should therefore be treated as a system dependent mechanism rather than a default explanation for improved NRA performance. Long distance H* migration remains debated in heterogeneous catalysis, especially on non-reducible supports or weak H binding surfaces under mild conditions. This uncertainty is amplified in electrocatalytic NRA, where electric fields, electrolyte ions, local pH gradients, nitrate and nitrite adsorption, and catalyst reconstruction can all reshape the interfacial hydrogen balance. Apparent spillover signatures may also arise from altered water dissociation kinetics, HER suppression, mass transport effects, or changes in adsorbate coverage. A credible assignment of hydrogen spillover therefore requires converging evidence from kinetic analysis, isotope labelling, *operando* spectroscopy, controlled poisoning experiments, and theoretical calculations.

### 4.1 WO_3_ color-change experiment

The WO_3_ color-change experiment is the oldest and most intuitive classical method, with its principle tracing back to Khoobiar's pioneering work in 1964.^100^ It has been demonstrated that yellow WO_3_ powder cannot spontaneously activate H_2_ or dissociate water. However, when physically mixed with a catalyst possessing hydrogen donor capability, H* migrates from donor sites to WO_3_, reducing it to deep-blue H_*x*_WO_3_. Navarro-Ruiz *et al.*^112^ observed a color change in WO_3_ in a mixed Pd/CNT and WO_3_ system, thereby corroborating hydrogen spillover. Dai *et al.*^113^ expanded upon this methodology, applying it to single-phase La_2_Sr_2_PtO_7+*δ*_ oxides. In NRA systems, the dynamic attenuation of the W=O stretching vibration peak (∼778 cm^−1^) is monitored by *in situ* Raman spectroscopy, thereby realizing an “electrochemical version of the WO_3_ color-change experiment.” However, the experiment is not equipped to provide quantitative data on migration distance, rate, and pathway. As a result, it is more appropriate to utilize the experiment as a preliminary screening tool.

#### 4.1.1 Radical trapping experiments

**Fig. 9:**
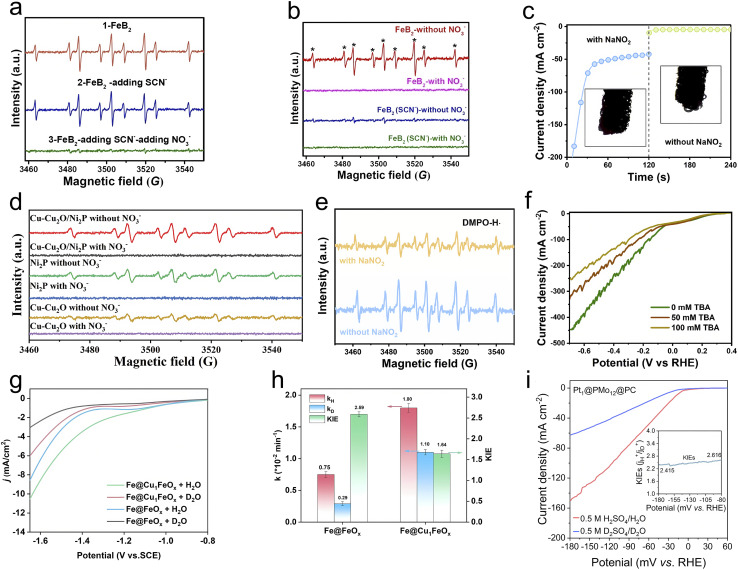
(a) EPR spectra of FeB_2_ with and without SCN^−^ poisoning under different conditions. (b) EPR spectra of FeB_2_ and FeB_2_ (SCN^−^) with and without NO_3_^−^. (c) EPR spectra of Cu–Cu_2_O/Ni_2_P, Ni_2_P, and Cu–Cu_2_O with and without NO_3_^−^. Copyright 2023, *Angewandte Chemie International Edition*. (d) Current density–time curves of the catalyst with and without NaNO_2_, demonstrating H* involvement in NO_2_^−^ reduction. (e) DMPO spin-trapping EPR spectra of collected electrolytes with and without NaNO_2_, confirming DMPO−H* adduct formation. (f) LSV curves at 0, 50, and 100 mM TBA concentrations. Copyright 2025, *Applied Catalysis B: Environment and Energy*. (g) LSV curves of Fe@Cu_1_FeO_*x*_ and Fe@FeO_*x*_ in H_2_O and D_2_O electrolytes. (h) Rate constants (*k*_H_, *k*_D_) and KIE values for Fe@FeO_*x*_ and Fe@Cu_1_FeO_*x*_. Copyright 2024, *Angewandte Chemie International Edition*. (i) Polarization curves of Pt_1_@_12_PMo_2_@PC in 0.5 M H_2_SO_4_/H_4_O and 0.5 M D_2_SO_4_/D_4_O; inset: potential-dependent KIE values. Copyright 2024, *Energy & Environmental Science*.

DMPO (5,5-dimethyl-1-pyrroline N-oxide) is the most commonly used spin-trapping agent. Upon binding with H*, it forms a DMPO-H adduct, which is subsequently detected by EPR. The presence of hydrogen radicals is indicated by a nine-peak EPR spectrum with a 1 : 1 : 2 : 1 : 2 : 1 : 2 : 1 : 2 : 1 : 1 intensity ratio in a nitrate-free electrolyte. Chu *et al.*^114^ employed a site-selective quenching strategy using thiocyanate (SCN^−^) to poison Fe sites of an FeB_2_ catalyst (Fig. 9a). The controlled design is particularly compelling, as evidenced by the presence of a discernible DMPO-H signal in a NO_3_^−^ free electrolyte. This signal undergoes a marked decrease or complete disappearance upon the addition of NO_3_^−^, thereby suggesting that H* is preferentially consumed during NO_3_^−^ reduction (Fig. 9b). As demonstrated in Fig. 9c, Cu–Cu_2_O/Ni_2_P exhibited the strongest DMPO-H signal in a NO_3_^−^-free electrolyte, followed by Ni_2_P and Cu–Cu_2_O. Upon the introduction of NO_2_^−^, the signals of all three catalysts disappeared completely,^115^ thereby confirming that surface-generated H is fully consumed in NO_3_^−^ reduction hydrogenation steps. In a Pt–Cu(OH)_2_/CF system,^116^*in situ* optical microscopy revealed that abundant hydrogen bubbles on the electrode surface nearly disappear upon NaNO_2_ addition (Fig. 9d), proving H* is efficiently consumed by NO_2_^−^. EPR characterization following DMPO addition provided further confirmation of this phenomenon. In the absence of NO_2_^−^, a nine-peak DMPO-H adduct signal was observed, whereas upon NO_2_^−^ addition, the signal was markedly attenuated (Fig. 9e). This result directly demonstrates that the NO_2_RR consumes substantial quantities of H*. *Tert*-butanol (*t*-BuOH) scavenging experiments provide complementary evidence: when *t*-BuOH is introduced, the NH_3_ yield and FE decline substantially (Fig. 9f), confirming H* as the core active species for NO_2_RR hydrogenation. A fundamental interpretative limitation remains: DMPO captures free H* in the solution phase, whereas the core of hydrogen spillover is the interfacial migration of surface-adsorbed H*. Consequently, alterations in the DMPO signal can only indirectly mirror surface H* consumption trends.

#### 4.1.2 Isotope labelling experiments

Deuterium isotope labelling is a standard method for identifying hydrogen-transfer steps. The underlying physicochemical basis of this phenomenon is attributable to the zero-point energy difference between the O–H and O–D bonds. The KIE (kH/kD) typically falls within the range of 2–7.^117^ In the event that the RDS involves H_2_O dissociation to generate H*, D_2_O substitution will lead to a significant rate decrease (KIE > 1). Ai *et al.*^118^ investigated Fe@Cu_1_FeO in a nitrate-free system. LSV measurements with D_2_O as the solvent showed that Fe@Cu_1_FeO still delivers a higher current density than Fe@FeO (Fig. 9g). The fitted KIE value of 1.64 was markedly lower than the 2.59 for Fe@FeO (Fig. 9h). This finding indicates that single-atom Cu effectively enhances water dissociation kinetics and promotes efficient H* spillover to adjacent Fe sites. Kang *et al.*^119^ conducted KIE experiments on Pt_1_@POMs@PC (Fig. 9i), with a KIE value of 2.4–2.6 indicating that hydrogen migration governs the HER rate.

### 4.2 Electrochemical characterization

#### 4.2.1 Tafel slope analysis

The Tafel slope acts as a classic kinetic tool to identify the rate-determining step of the HER and NRA.^120^ Slopes below 30 mV dec^−1^ are generally attributed to the Volmer-diffusion-Tafel mechanism, where surface diffusion of H* dominates the reaction rate.^121,122^ Pt2Ir1/CoP and Ru1Fe1/CoP catalysts deliver slopes of 25.2 and 24.5 mV dec^−1^, matching this model well.^123^ Nevertheless, this judgment cannot be directly applied to NRA systems. A Tafel slope below 30 mV dec^−1^ may originate from effects irrelevant to hydrogen spillover, such as mass transport restrictions inside porous catalyst layers, potential-induced shifts in adsorbate coverage, and overlapping parallel reaction channels in multi-step electrocatalysis.^124^ Since NRA follows a complicated multi-step reaction sequence, its low apparent Tafel slope usually originates from multiple coupled elementary steps instead of one single rate-limiting stage. Accordingly, Tafel fitting results can only serve as auxiliary corroboration and are insufficient to independently confirm the existence of hydrogen spillover.

#### 4.2.2 Cyclic voltammetry (CV)

**Fig. 10:**
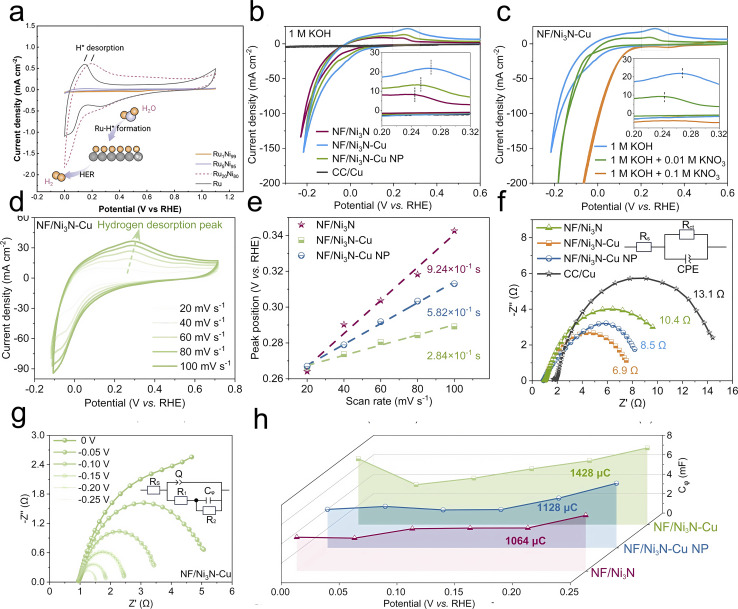
(a) Cyclic voltammetry (CV) curves of Ru, Ru_1_Ni_99_, Ru_5_Ni_95_, and Ru_20_Ni_80_ in 0.1 M KOH, illustrating H* desorption and Ru–H* formation regions. (b) LSV curves of NF/Ni_3_N, NF/Ni_3_N–Cu, NF/Ni_3_N–Cu NP, and CC/Cu in 1 M KOH; inset: enlarged hydrogen desorption peak region. (c) LSV curves of NF/Ni_3_N–Cu in 1 M KOH, 1 M KOH + 0.01 M KNO_3_, and 1 M KOH + 0.1 M KNO_3_; inset: enlarged hydrogen desorption peak region. (d) CV curves of NF/Ni_3_N–Cu at scan rates from 20 to 100 mV s^−1^. (e) Hydrogen desorption peak position *vs.* scan rate plots for NF/Ni_3_N, NF/Ni_3_N–Cu, and NF/Ni_3_N–Cu NP, with fitted hydrogen desorption time constants. (f) Nyquist plots of NF/Ni_3_N, NF/Ni_3_N–Cu, NF/Ni_3_N–Cu NP, and CC/Cu; inset: equivalent circuit. (g) Nyquist plots of NF/Ni_3_N–Cu at potentials from 0 to −0.25 V *vs.* RHE; inset: equivalent circuit. (h) *Q*_H_ and double-layer capacitance (*C*_dl_) of NF/Ni_3_N–Cu, NF/Ni_3_N–Cu NP, and NF/Ni_3_N at different potentials. Copyright 2025, *Angewandte Chemie International Edition*.

CV is capable of detecting the desorption behavior of surface-adsorbed H*. Fan *et al.*^125^ revealed the quantitative regulation of surface H* coverage by Ru content in RuNi alloys (Fig. 10a). Xiong *et al.*^84^ established a direct correlation between H* coverage and the spillover effect in the NF/Ni_3_N–Cu system. The H* desorption peak area in the hydrogen underpotential deposition (H_upd_) region was markedly larger than that of the controls (Fig. 10b), and the peak area decreased monotonically with increasing NO_3_^−^ concentration (Fig. 10c). However, CV signals in the H_upd_ region should be interpreted with caution in NRA electrolytes. Nitrate, nitrite, and other NO_*x*_ intermediates can change the local interfacial environment, shift H adsorption or desorption peak positions, and alter peak areas through competitive adsorption or surface reconstruction. Therefore, changes in the H_upd_ peak area or peak position provide indirect information on surface H coverage and H transfer kinetics, rather than direct proof of hydrogen spillover. Although pristine NF/Ni_3_N shows strong water dissociation capability, its NRA activity is lower than that of NF/Ni_3_N–Cu. This comparison indicates that H does not efficiently migrate to Cu sites and is mainly lost through the HER. Furthermore, variable scan rate CV (20–100 mV s^−1^) analysis demonstrated that the slope of the H desorption peak potential *versus* log *v* for NF/Ni_3_N–Cu is significantly lower than that for NF/Ni_3_N (Fig. 10d). This finding indicates that H* spilled over to the Cu surface exhibits a reduced desorption overpotential and accelerated desorption kinetics (Fig. 10e and f). This acceleration effect provides kinetic support for facilitated H transfer from Ni_3_N to Cu, but it should be considered together with isotope labelling, *operando* spectroscopy, and theoretical calculations before assigning a hydrogen spillover pathway.

### 4.3 Electrochemical impedance spectroscopy (EIS)

The EIS facilitates the real-time observation of adsorption and desorption kinetics.^126–128^ Xiong *et al.*^84^ established a direct scaling relationship between the hydrogen adsorption charge (*Q*_H_) and spillover efficiency. Nyquist plots collected at different potentials (Fig. 10g) were fitted using a dual-parallel equivalent circuit model to extract the hydrogen adsorption pseudocapacitance. The integral of the overpotential (*Q*_H_) was then obtained. The NF/Ni_3_N–Cu specimen demonstrated the highest QH of 1428 µC, which is 1.67 times greater than the QH of CC/Cu (855 µC). The significant disparity in the QH difference between Ni_3_N–Cu NP (1128 µC) and Ni_3_N–Cu (1428 µC), with interfacial contact intimacy operating as the exclusive variable, underscores the notion that enhancing interfacial contact leads to an augmentation in H* transport efficiency through Ni–N–Cu bridging bonds. The charge transfer resistance *R*_ct_ was also significantly lower for NF/Ni_3_N–Cu (Fig. 10h), confirming that intimate interfacial contact simultaneously accelerates electron transport and H* migration.

### 4.4 *In situ* spectroscopic characterization

#### 4.4.1 ATR-IR and ATR-SEIRAS

**Fig. 11:**
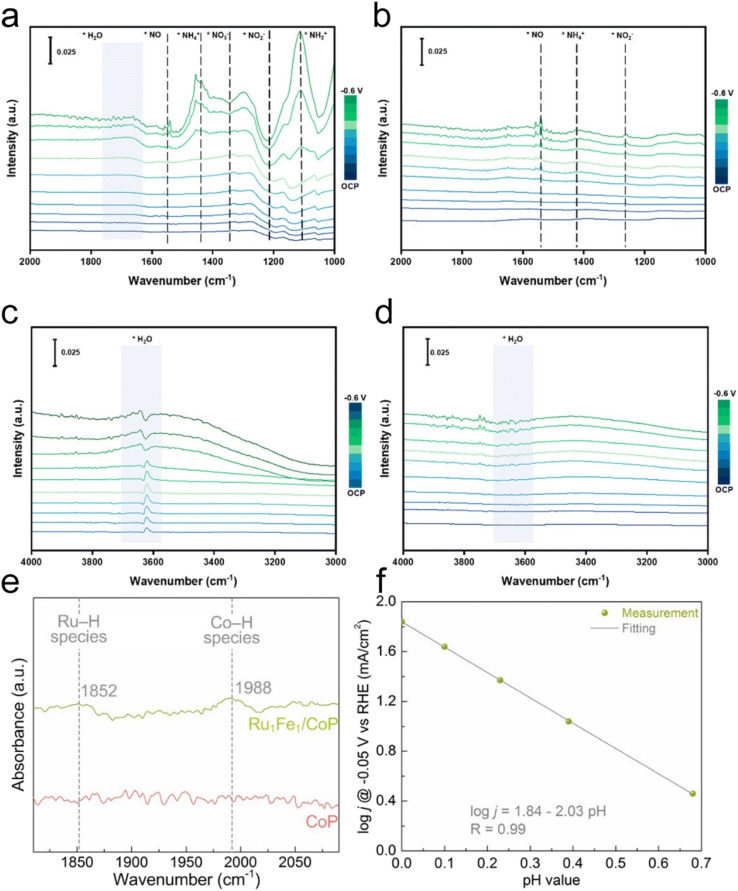
*In situ* ATR-SEIRAS spectra of NO_3_RR intermediates (*H_2_O, *NO, *NH_4_^+^, *NO_3_^−^, *NO_2_^−^, and *NH_2_^+^) on the catalyst (a) with and (b) without NO_3_^−^, recorded from OCP to −0.6 V *vs.* RHE in the 1000–2000 cm^−1^ region. *In situ* ATR-SEIRAS spectra in the O–H stretching region (3000–4000 cm^−1^) showing interfacial *H_2_O evolution on the catalyst (c) with and (d) without NO_3_^−^ from OCP to −0.6 V *vs.* RHE. Copyright 2025, *Green Chemistry*. (e) *Operando* ATR-IR spectra of CoP and Ru_1_Fe_1_/CoP in Ar-saturated 0.5 M H_2_SO_4_, showing Ru–H (1852 cm^−1^) and Co–H (1988 cm^−1^) surface hydride species. (f) Plot of log *j* at −0.05 V *vs.* RHE as a function of pH for Ru_1_Fe_1_/CoP (slope: −2.03, *R* = 0.99), indicating second-order proton dependence. Copyright 2022, *ACS Energy Letters*.

*In situ* attenuated total reflection infrared spectroscopy (ATR-IR) can directly detect metal–hydrogen (M–H) bond stretching vibrations, thereby demonstrating real-time H* generation at the donor sites. Attenuated total reflection surface-enhanced infrared absorption spectroscopy (ATR-SEIRAS) can enable the concurrent detection of H* signals at both the donor and acceptor sites.^129–132^ Li *et al.*^60^ employed *in situ* ATR-SEIRAS on Cu_5_–Co_5_ to trace the complete NRA pathway: the spectra sequentially detected *NO_3_^−^ (1350 cm^−1^), *NO_2_^−^ (1220 cm^−1^), *NO (1550 cm^−1^), *NH (1280 cm^−1^), *NH_2_ (1150 cm^−1^), and NH_4_^+^ (1440 cm^−1^) (Fig. 11a). In the pure Cu control, the *NO_2_^−^ and *NO peak intensities were markedly higher (Fig. 11b), indicating weaker capacity for NO_3_^−^ hydrogenation and NO_3_^−^ accumulation as the primary by-product. The interfacial water O–H stretching vibration near 3600 cm^−1^ shifted to lower wavenumbers on Cu_5_–Co_5_ but remained nearly unchanged on pure Cu (Fig. 11c and d), indicating Co-site-promoted water dissociation. Qu *et al.*^126^ employed ATR-IR to directly confirm hydrogen spillover in Ru_1_Fe_1_/CoP. The analysis revealed the presence of strong characteristic peaks at 1992 cm^−1^ (Ru–H) and 1858 cm^−1^ (Co–H). In contrast, bare CoP exhibited no distinct peaks near 2000 cm^−1^ (Fig. 11e), thereby confirming H* spillover from Ru_1_Fe_1_ sites to the CoP support. In addition, the calculated reaction order of ∼2.03 and the H adsorption Gibbs free energy of 20.31 kJ mol^−1^ (Fig. 11f) are in close agreement with theoretical predictions for catalysts operating *via* a hydrogen spillover mechanism. ATR-IR is particularly suited for real-time tracking of hydrogen and intermediate evolution. It is often combined with electrochemical measurements, DFT calculations, and *in situ* Raman spectroscopy to achieve comprehensive mechanistic elucidation.

#### 4.4.2 *Operando*/*in situ* Raman spectroscopy

**Fig. 12:**
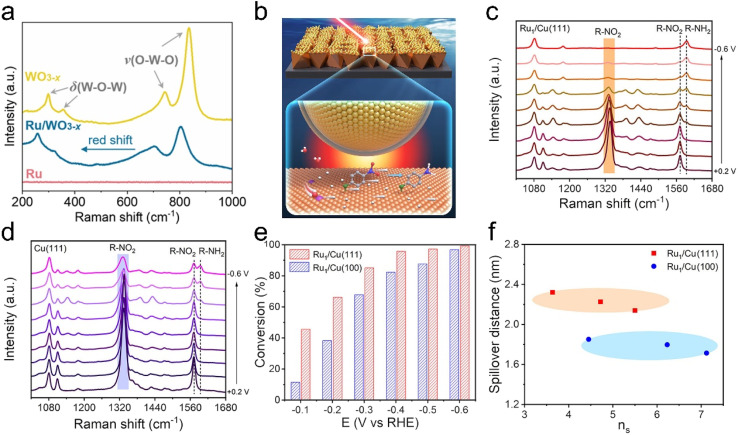
(a) Raman spectra of Ru, WO_3−*x*_, and Ru/WO_3−*x*_, showing characteristic *ν*(O–W–O) and *δ*(W–O–W) modes and red shift upon Ru loading. Copyright 2026, *Nature Communications*. (b) Schematic illustration of the *in situ* SHINERS setup for monitoring NRA intermediates. (c and d) *In situ* SHINERS spectra of NRA from +0.2 to −0.6 V *vs.* RHE on (c) Ru_n_/Cu(111) and (d) Cu(111), showing evolution of R–NO and R–NH_2_ intermediates. (e) NO_3_^−^ conversion as a function of applied potential for Ru_*n*_/Cu(111) and Ru_*n*_/Cu(100). (f) Hydrogen spillover distance as a function of Ru site density (*n*_s_) on Ru_*n*_/Cu(111) and Ru_*n*_/Cu(100). Copyright 2019, *Journal of the American Chemical Society*.

*In situ* Raman spectroscopy can facilitate the tracking of lattice hydrogen intercalation–deintercalation dynamics.^133^ Liang *et al.*^93^ employed *in situ* Raman on Ru/WO_3−*x*_: upon Ru loading, W–O–W and O–W–O vibrations underwent a distinct redshift (Fig. 12a), indicating strong metal–support interaction (SMSI) with electron transfer from Ru to the WO_3−*x*_ support. The resulting electron deficiency on Ru creates Lewis acidic sites favorable for NO_3_^−^ adsorption, while electron enrichment on WO_3−*x*_ promotes H^+^ intercalation and H_*x*_WO_3_ bronze-phase formation, providing the thermodynamic basis for reverse H spillover to Ru sites. Li *et al.*^134^ achieved nanoscale quantification of spillover distance using SHINERS combined with *para*-nitrothiophenol (pNTP) probe molecules self-assembled alongside SHINs nanoparticles on the electrode surface (Fig. 12b). The Raman peak at 1571 cm^−1^ (pNTP benzene ring vibration) underwent a gradual shift to 1594 cm^−1^ as the cathodic potential underwent negative movement, corresponding to the hydrogenation product pATP (Fig. 12c and d). On Ru_1_/Cu(111) and Ru_1_/Cu(100), the pATP signal emerged at −0.1 V *vs.* RHE, whereas pristine Cu required −0.4 V, thereby directly confirming H spillover from Ru_1_ sites to the Cu surface. The integration of the 1337 cm^−1^ peak area demonstrated that Ru_1_/Cu(111) exhibited superior performance in comparison to Ru_1_/Cu(100) across the entire potential range, particularly within the −0.1 to −0.3 V region (Fig. 12e). By adjusting the Ru site density, the spillover distance was determined to be approximately 2.0–2.4 nm on Cu(111) and 1.6–1.8 nm on Cu(100) (Fig. 12f). This advancement transformed hydrogen spillover from a theoretically inferred phenomenon to a quantitatively measurable experimental observable, thereby establishing a SHINERS paradigm for investigating the crystal-facet dependence of spillover distance.

#### 4.4.3 *In situ* XAS

**Fig. 13:**
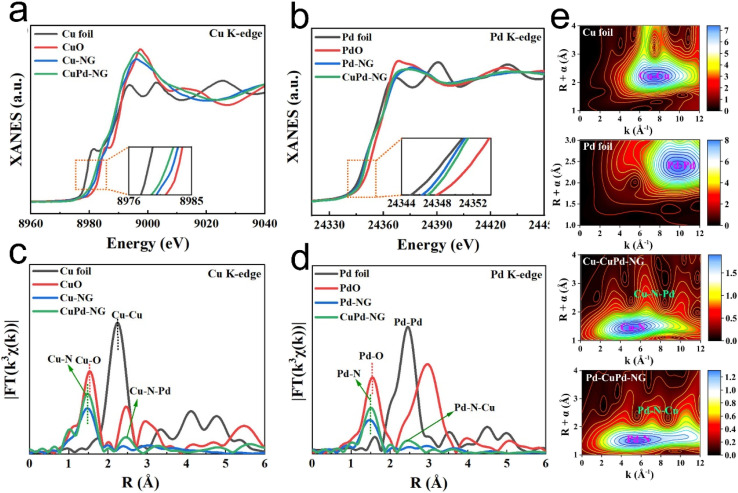
XANES spectra at the (a) Cu K-edge and (b) Pd K-edge of Cu foil, CuO, Cu-NG, Pd foil, PdO, Pd-NG, and CuPd-NG; insets: enlarged near-edge regions showing oxidation state shifts. Fourier-transformed EXAFS spectra at the (c) Cu K-edge and (d) Pd K-edge, showing characteristic Cu–N, Cu–O, Cu–Cu, Cu–N–Pd, Pd–N, Pd–O, Pd–Pd, and Pd–N–Cu scattering paths. (e) Wavelet transform (WT) EXAFS contour plots of Cu foil, Pd foil, Cu in CuPd-NG, and Pd in CuPd-NG, confirming Cu–N–Pd and Pd–N–Cu coordination environments. Copyright 2025, *ACS Catalysis*.

X-ray absorption spectroscopy (XAS) provides an independent verification dimension from the electronic-structure perspective.^135–137^ XANES can facilitate the tracking of valence-state changes at donor metal sites during reactions. An increase in the oxidation state increases near the water dissociation potential, followed by a recovery at the NO_*x*_ hydrogenation potential, serves as an indication of the formation of a dynamic redox cycle with H* generation and consumption at the donor site. Zang *et al.*^82^ employed XAS on CuPd-NG: Cu K-edge XANES demonstrated a shift in the Cu absorption edge to lower energy by approximately 0.5 eV (Fig. 13a), while Pd shifted to higher energy (Fig. 13b). This directional charge results in the formation of a weak BEF within the Pd–N–Cu bridge bond, thereby driving directional H* migration. EXAFS analysis confirmed the atomistic dispersion of both Pd and Cu, ruling out any possibility of metal–metal coordination (Fig. 13c and d). The wavelet transforms (WT) further elucidated the metal–N and metal–metal coordination pathways (Fig. 13e).

### 4.5 Theoretical calculations

#### 4.5.1 DFT free-energy calculations

**Fig. 14:**
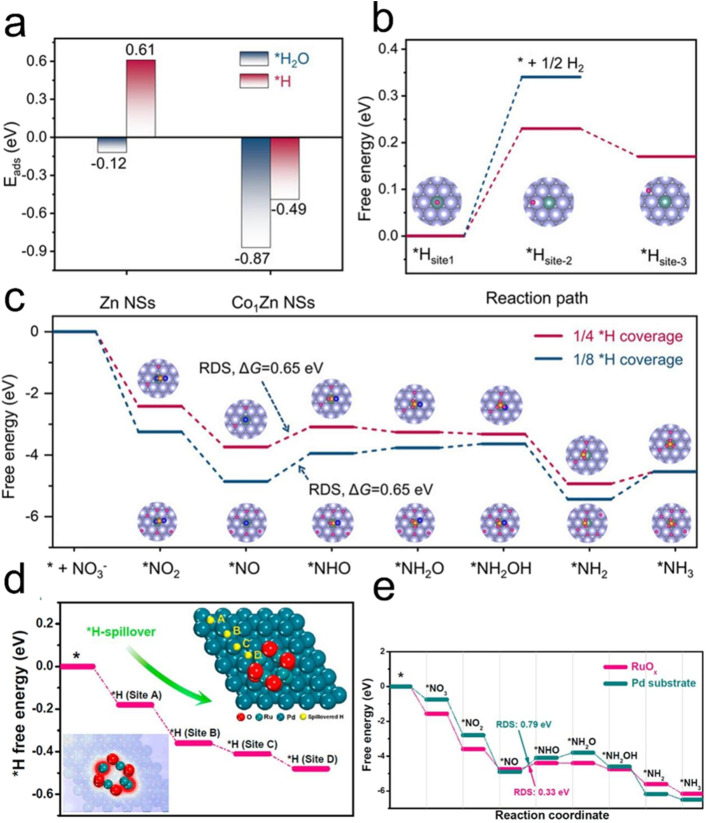
(a) Adsorption energies of *H_2_O and *H on Zn NSs and Co_1_Zn NSs. (b) Free energy diagram of water dissociation and *H migration across three adsorption sites (H_site-1_ → H_site-2_ → H_site-3_) on Co_1_Zn NSs. (c) Gibbs free energy diagrams of the NO_3_RR on Co_1_Zn NSs under 1/4 and 1/8 *H surface coverage, with the rate-determining step (RDS, Δ*G* = 0.65 eV) identified at the *NO → *NHO step. Copyright 2025, *Angewandte Chemie International Edition*. (d) *H free energy diagram illustrating spontaneous H spillover from Ru to Pd sites (A → B → C → D) on RuO_*x*_/Pd; insets: top view of the H spillover pathway and interfacial charge density distribution. (e) Gibbs free energy diagrams of NRA on RuO_*x*_ and the Pd substrate, showing RDS values of 0.79 eV (RuO_*x*_) and 0.33 eV (Pd substrate). Copyright 2023, *ACS Nano*.

Computational modeling is a convenient and efficient approach for elucidating reaction mechanisms and establishing activity descriptors.^138^ Zeng *et al.*^91^ employed DFT calculations on Co_1_Zn. Furthermore, the adsorption energies of H_2_O and H* on CoZn(002) are more negative than those on Zn(002) (Fig. 14a). The H* spillover barrier from the Co site to adjacent Zn sites (0.23 eV) is lower than the H_2_ formation barrier (0.34 eV) (Fig. 14b), which thermodynamically confirms that spillover is favored over the HER. In the absence of atomic dispersion (Co_43_/Zn_57_), H* is preferentially consumed *via* the HER, thereby demonstrating that atomic dispersion is a necessary condition for the “hydrogen pump” function. As the spillover-driven H* coverage increases from 1/8 ML to 1/4 ML, the RDS free-energy barrier is substantially reduced (Fig. 14c).

Chu *et al.*^72^ employed DFT on RuO_*x*_/Pd, revealing that the H_2_O dissociation barrier on Pd (0.52 eV) is significantly lower than that on graphene (1.41 eV). Stepwise H* adsorption free energies (Fig. 14d) revealed that H* adsorption strength progressively increases along the Pd → Ru_*x*_/Pd interface direction, with the electron-rich interface acting as an “electron reservoir.” The hydrogen-bond-mediated hydrogenation pathway exhibits a barrier of only 0.33 eV, which is significantly lower than the 0.79 eV barrier of the conventional pathway (Fig. 14e).

#### 4.5.2 PDOS and charge density difference analysis

**Fig. 15:**
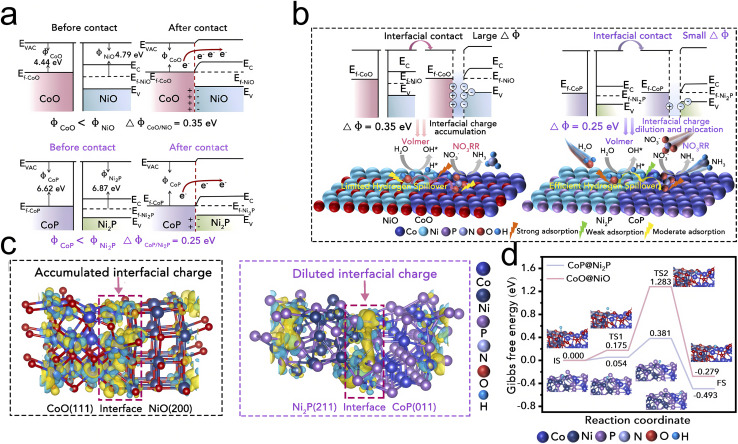
(a) Energy band diagrams of CoO/NiO and CoP/Ni_2_P before and after contact, showing work function differences of Δ*Φ*_CoO/NiO_ = 0.35 eV and Δ*Φ*_CoP/Ni2P_ = 0.25 eV. (b) Schematic illustration of the effect of Δ*Φ* on interfacial hydrogen spillover: large Δ*Φ* (CoO@NiO, 0.35 eV) leads to interfacial charge accumulation and limited hydrogen spillover, while small Δ*Φ* (CoP@Ni_2_P, 0.25 eV) results in interfacial charge dilution and relocation, enabling efficient hydrogen spillover for NRA. (c) Charge density difference maps of CoO(111)/NiO(200) (accumulated interfacial charge) and Ni_2_P(211)/CoP(011) (diluted interfacial charge) interfaces (yellow: charge accumulation; blue: charge depletion). (d) Gibbs free energy diagrams of *H spillover from Ni to Co sites on CoP@Ni_2_P and CoO@NiO, with transition states (TS1 and TS2) and final state (FS) identified. Copyright 2025, *Nano Energy*.

Projected density of states (PDOS) analysis reveals the influence of electronic-structure differences on H* migration directionality by comparing d-band center positions (*ε*_d_). In a recent study, Wang *et al.*^52^ employed a combination of UPS experiments and DFT on CoP@Ni_2_P: UPS measurements revealed that the phosphidized Δ*Φ* is 0.25 eV, which is significantly smaller than the 0.35 eV observed before phosphidation (Fig. 15a). DFT calculations reveal that the work functions of CoP(001) and Ni_2_P(001) are 6.62 eV and 6.87 eV, respectively (Fig. 15b). Charge density difference maps revealed pronounced charge depletion at the CoP/Ni_2_P interface (Fig. 15c), facilitating cross-interface H* permeation. The comprehensive energy profile for H* migration (Fig. 15d) showed that the barrier decreased from approximately 1.283 eV (CoO@NiO) to 0.381 eV (CoP@Ni_2_P) following phosphidation, thereby providing quantitative guidance for the design of work-function-engineered spillover catalysts.

#### 4.5.3 Molecular dynamics simulations

**Fig. 16:**
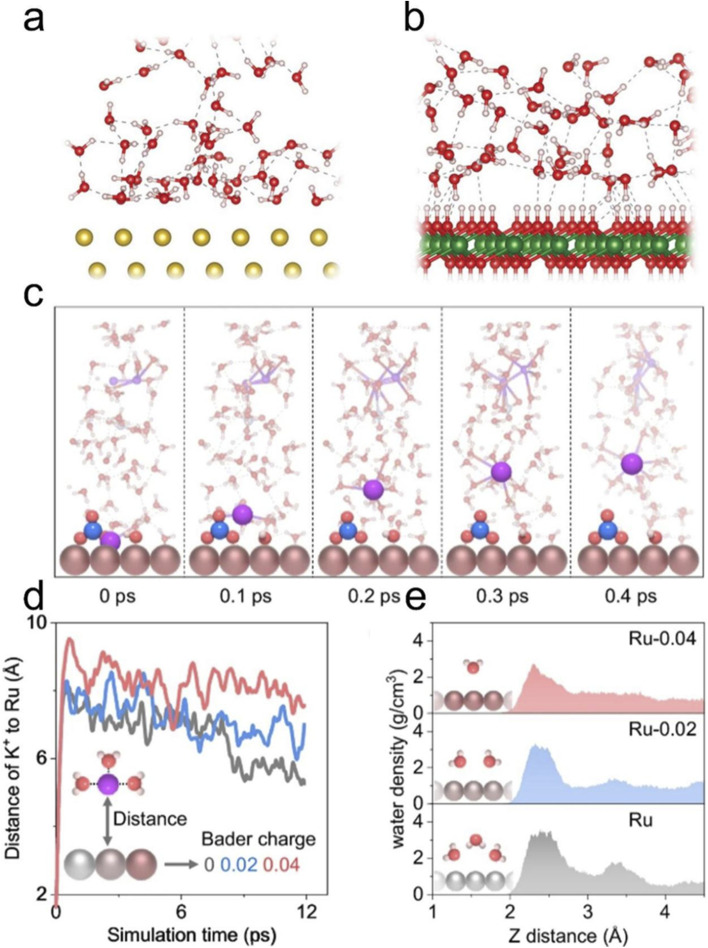
Snapshots of interfacial water structures from AIMD simulations on (a) bare Ru(001) and (b) Ni(OH)_2_-covered Ru surfaces; Ru, Ni, O, and H atoms are shown in yellow, green, red, and white, respectively; gray dashed lines denote hydrogen bonds. Copyright 2025, *Advanced Materials*. (c) Sequential AIMD snapshots (0–0.4 ps) illustrating the dynamic adsorption of K^+^ approaching the Ru-0.04 surface, with water molecules and NO_3_^−^ shown in the electrolyte layer. (d) Time evolution of K^+^-to-Ru distance during AIMD simulations on Ru (Bader charge = 0), Ru-0.02, and Ru-0.04 surfaces, showing accelerated K^+^ adsorption with increasing surface charge density; inset: schematic of K^+^ distance measurement. (e) Water density profiles along the *Z*-direction for Ru, Ru-0.02, and Ru-0.04 surfaces, showing modulation of interfacial water arrangement with increasing Bader charge; insets: representative interfacial water orientations. Copyright 2024, *Nature Communications*.

MD simulations are a computational method that facilitates the integration of static DFT energetics with *in situ* experimental observations. This integration is achieved by the simulation of thermal fluctuations, solvation effects, and the dynamic evolution of interfacial water hydrogen bond networks.^139–141^ Lv *et al.*^100^ employed AIMD on Ru–Ni(OH)_2_. On pristine Ru, interfacial water is loosely arranged, whereas the surface –OH groups of Ni(OH)_2_ anchor and polarize adjacent water molecules through strong hydrogen bonds, rendering the interfacial water layer more compact (Fig. 16a and b). Li *et al.*^104^ employed AIMD on 2D electron deficient Ru/NC, observing that hydrated K^+^ can detach from the Ru surface within 0.4 ps (Fig. 16c). Concurrently, NO_3_^−^ and OH^−^ become enriched at the interface (Fig. 16d and e). This exclusion effect exhibits cationic generality (Li^+^, Na^+^, and Cs^+^), with K^+^ delivering the highest NH_3_ yield. The resulting loose interfacial water layer simultaneously suppresses the HER and accelerates NO_3_^−^ mass transport, thereby enabling an NH_3_ yield of 55.4 mg cm^−2^ h^−1^ at 1 A cm^−2^ with FE remaining stable at 90–94% for over 120 hours.

#### 4.5.4 Quantitative descriptors for spillover efficiency

Although the methods discussed above provide useful evidence for hydrogen spillover, many of them remain qualitative or semi quantitative. A more rigorous comparison across NRA catalysts requires operational metrics that connect H* generation, interfacial transport, and NO_*x*_ hydrogenation. We suggest that future studies report an apparent spillover efficiency, defined as the fraction of donor generated H* that is transferred to reduction sites and consumed in nitrate hydrogenation rather than lost through the HER. In practice, this value may be estimated by combining isotope labelling, calibrated electrochemical H adsorption measurements, transient kinetic analysis, and *operando* spectroscopic probes.

Several complementary descriptors can be used to quantify different aspects of spillover. The isotope-derived H transfer fraction can distinguish transferred H* from locally generated or solvent supplied hydrogen in the final NH_3_ product. The apparent H* transfer rate constant can be extracted from transient current, isotope switching, or time resolved spectroscopic measurements. The effective spillover distance can be estimated using spatially resolved probe reactions or surface enhanced spectroscopies. The nitrate-induced attenuation of surface H coverage, obtained from calibrated CV, EIS, or *operando* spectroscopy, can provide an indirect measure of how efficiently H* is redirected from the HER toward NO_*x*_ hydrogenation. These descriptors should be reported together with nitrate concentration, electrolyte composition, pH, applied potential, current density, catalyst loading, and reactor configuration.^142–146^ Such standardized reporting would make spillover efficiency comparable across different catalyst architectures and would reduce the risk of assigning improved NRA activity to spillover based only on enhanced NH_3_ yield or FE.

## 5. Conclusion and outlook

### 5.1 Conclusion

This review has systematically examined the role of hydrogen spillover in NRA across its fundamental mechanism, characterization techniques, and catalyst design strategies. The initial phase of the study involved the establishment of the mechanistic principles and key preconditions. It was determined that donor sites activate water, thereby generating H* in a continuous manner. Reduction sites were found to adsorb NO_3_^−^ and convert NO_*x*_ intermediates. Interfacial migration channels were identified as the means of directional H* transport. The spatial decoupling of water dissociation from substrate hydrogenation disrupts the zero-sum competition between the HER and NRA that is intrinsic to single-site catalysts. Utilizing this framework as a foundation, a comprehensive survey was conducted on catalyst design strategies across distinct dimensions: electronic structure regulation, interfacial coupling, migration direction control, migration pathway shortening, and interfacial microenvironment engineering. This survey methodically illustrates the potential and design principles of each strategy through detailed case studies. Concurrently, a multi-dimensional cross-validation framework was established for confirming the occurrence of hydrogen spillover, encompassing four categories of validation methodologies: experimental verification, electrochemical analysis, *in situ* spectroscopic characterization, and theoretical calculations. Notwithstanding the substantial progress that has been made in recent years, the field continues to face significant challenges. Consequently, we hereby put forward the following specific directions for future research.

### 5.2 Outlook

#### 5.2.1 Establishing quantitative metrics and integrating isotope-labelled *operando* tools

Despite the mounting body of experimental evidence for hydrogen spillover in electrocatalytic NRA, concerns regarding its credibility and reproducibility persist due to the absence of quantitative metrics that facilitate cross-system comparison. In the future, endeavors should prioritize the comprehensive integration of isotope-labeled *operando* tools. This involves the utilization of D_2_O/H_2_O mixed electrolytes to trace the origin and migration trajectory of H*, thereby establishing quantitative correlations between product distributions and reduction pathways. Additionally, the development of isotope-dependent Tafel analysis methods is crucial for quantifying H* migration kinetics. Furthermore, the synchronization of *in situ* Raman spectroscopy with *operando* XAS is essential for the dynamic monitoring of reaction intermediate evolution and the spatiotemporal coupling with valence-state changes at donor sites. Concurrently, complementary analytical techniques, including ion chromatography, the indophenol blue method, and NMR, should be adopted to cross-validate NH_3_ yield. Gas diffusion electrodes should be employed to enhance mass transport, and ATR-SEIRAS, Raman spectroscopy, and AIMD simulations should be integrated to elucidate the synergistic roles of interfacial water structure, hydrogen-bond networks, and hydrogen spillover in directing reaction pathways.

#### 5.2.2 Extending hydrogen spillover to non-precious-metal catalysts

The present applications of hydrogen spillover in NRA are predominantly concentrated on Pt, Pd, and Ru precious-metal catalysts. The intrinsically high H* generation capacity of these catalysts focuses the design challenge on regulating H* migration to suppress the HER. In contrast, non-precious-metal systems necessitate the deliberate “manufacturing” of the hydrogen-supply function and a clear division of labor among components. The merits of non-precious metals are evident in their highly tunable electronic structure, which can be modulated through alloying, doping, or support engineering to adjust the d-band center. Additionally, their design flexibility, attributable to multi-component cooperativity, is a notable advantage. These metals are not only cost-effective but also environmentally compatible, further enhancing their appeal and potential applications. The initial success of Co_1_Zn single-atom alloys and Ni_3_N-based systems suggests that precise coordination-environment engineering can narrow the gap with precious metals. Future research endeavors should prioritize the establishment of universal design principles for non-precious-metal catalysts, informed by a more profound mechanistic understanding of hydrogen spillover. These efforts should culminate in the development of high-performance catalytic systems that exhibit minimal precious-metal loading, or preferably, are entirely free of precious metals.

#### 5.2.3 Prioritizing scalability and industrial feasibility

At the application level, NRA should be evaluated primarily as a nitrate abatement and reactive nitrogen recovery technology, not as a standalone substitute for Haber–Bosch ammonia production from atmospheric N_2_. Real nitrate-containing wastewaters differ substantially from the simplified electrolytes used in most NRA studies. Chloride, sulfate, carbonate, phosphate, hardness ions, and dissolved organic matter can reshape the local reaction environment before nitrate reduction occurs. These species may compete with nitrate or NO_*x*_ intermediates for interfacial sites, modify the hydrogen bond network of interfacial water, buffer local pH, or block H donor and H acceptor sites. Chloride can also promote side reactions or accelerate corrosion under electrochemical polarization. Organic species may adsorb on metal or oxide surfaces and suppress water dissociation. These effects can change H* coverage, H* residence time, and the driving force for interfacial H* migration. A spillover pathway verified in purified nitrate electrolyte may therefore not operate with the same efficiency in real wastewater. Future studies should evaluate hydrogen spillover catalysts in matrix controlled electrolytes and real wastewater samples, with parallel monitoring of nitrate conversion, NH_3_ selectivity, HER activity, catalyst reconstruction, and surface poisoning.

Most reported hydrogen spillover NRA systems have been evaluated in H type cells, where catalysts are exposed to bulk aqueous electrolyte and mass transport is relatively slow. Flow cells and MEA zero gap devices create a different interfacial environment. Nitrate delivery, water activity, ionomer coverage, local electric field, product removal, and gas bubble management can all affect H* generation and migration. A shorter electrolyte distance can improve current density, while also changing local pH, NO_*x*_ coverage, and the balance between NRA and the HER. For spillover catalysts, the contact among donor sites, acceptor sites, the ionomer, and the membrane becomes especially important. H* residence time and interfacial transfer pathways may differ from those in bulk aqueous electrolytes. Future studies should examine spillover catalysts under flow cell and MEA conditions, with attention to from the difference insingle pass nitrate conversion, NH_3_ recovery, energy efficiency, membrane crossover, and long term interface stability. Beyond electrolyte matrix effects, the practical industrial translation of spillover catalysts also requires systematic advances in reaction efficiency, catalyst durability and large-scale electrolysis hardware.

Beyond catalyst activity, practical implementation requires several engineering constraints to be evaluated together. Real wastewater operation requires tolerance to competing ions, organic matter, hardness ions, and fluctuating nitrate concentrations. High current density operation requires sufficient nitrate delivery, controlled local pH, stable water activity, and rapid removal of NH_3_ or NH_4_^+^ from the electrode vicinity. Flow cells and MEA devices further introduce issues of membrane crossover, salt accumulation, electrode flooding or drying, gas bubble management, and ionomer coverage of active sites. These factors can change H coverage, H residence time, and interfacial H* transfer, thereby altering the balance between NRA and the HER. Long term operation also requires resistance to catalyst dissolution, surface reconstruction, donor site deactivation, and poisoning by inorganic or organic impurities. Future studies should therefore report not only FE and NH_3_ yield, but also single pass nitrate conversion, NH_3_ recovery efficiency, energy consumption for product separation, membrane stability, and continuous operation time under realistic wastewater conditions.

In order to advance toward practical application, efforts should be concentrated on the following aspects. (a) Enhancing reaction rate and energy efficiency: the driving force for hydrogen spillover originates from the difference in Δ*G*_H*_ between sites; however, an excessively large difference in Δ*G*_H*_ serves to increase the spillover barrier. The efficacy of prevailing strategies, encompassing interfacial water regulation, local alkalinity enhancement, and solid–liquid interface engineering, has been demonstrated in mitigating HER competition and mass-transport limitations. Further exploration of synergistic effects among these strategies is anticipated to yield additional breakthroughs. (b) Enhancing catalyst stability: hydrogen spillover systems necessitate meticulous consideration of the deactivation of donor sites under reaction conditions. The principal deactivation pathways include metal dissolution and leaching, surface reconstruction, active-site poisoning, and particle migration and aggregation driven by the evolution of metal–support interactions. The construction of protective layers, precise anchoring and spatial confinement, alloying, and high-entropy alloy design are viable strategies for enhancing stability. (c) The transition from the laboratory to practical electrolysis systems: the limitations of H-type cells are attributed to their mass-transport efficiency and product accumulation. Flow cells, equipped with gas diffusion electrodes that establish a gas–liquid–solid three-phase interface, can enhance current density and reaction stability to a considerable extent. In addition, the optimization of energy consumption in product separation processes, the integration of an electrocatalytic reactor with an acid-absorption chamber to facilitate continuous NH_3_ collection, and the execution of comprehensive economic feasibility assessments are imperative steps that must be pursued concurrently for large-scale implementation.

#### 5.2.4 Theoretical modeling of hydrogen spillover in NRA

Computational analysis is imperative for elucidating the reaction mechanism, electronic structure, and kinetic trends of hydrogen-spillover-enhanced NRA. AIMD simulations have revealed how interfacial water structure regulation lowers the Volmer barrier. They have also clarified how cation exclusion suppresses the HER. DFT calculations provide thermodynamic criteria for designing adsorption energy gradients between donor and acceptor sites. Microkinetic modeling is a scientific framework that integrates the principles of DFT energetics with macroscopic reaction rates and product distributions. This integration enables a comprehensive understanding of the relationship between the microscopic and macroscopic scales in chemical reactions, facilitating the prediction of material properties and processes. However, contemporary DFT calculations predominantly depend on static minimum-energy paths under vacuum conditions, with inadequate consideration of solvation effects, electrode potential, and electric fields. Spillover transition-state searches are contingent on the NEB method and are computationally onerous. The development of constant-potential DFT methods and machine-learning-potential-accelerated MD simulations to directly model the full process of H* generation, migration, and hydrogenation at the solid–liquid interface under realistic electrochemical conditions is an urgent direction for theoretical modeling.

#### 5.2.5 Machine-learning-accelerated high-throughput screening

The development of high-performance electrocatalysts is predominantly characterized by a trial-and-error approach, underscoring the need for well-defined design principles that can facilitate more systematic and efficient processes. This review has cataloged multiple descriptors associated with hydrogen-spillover-enhanced NRA activity, including Δ*G*_H*_, work function difference, d-band center, interfacial charge transfer, and spillover distance. However, the interactions among these descriptors and their synergistic contributions to overall performance have not been systematically quantified. The integration of machine-learning-assisted high-throughput screening methodologies to construct a multi-descriptor prediction framework has the potential to enhance the efficiency and precision of catalyst discovery processes. The following key steps must be followed to ensure the success of this endeavor: (1) the construction of a diverse catalyst database encompassing donor sites, acceptor sites, and interfacial bridging architectures is essential. (2) Multi-descriptor cooperative screening is necessary to identify the descriptor combination with the highest predictive power. (3) Multitask prediction models must be developed to simultaneously optimize NH_3_ yield, FE, and stability. (4) Closed-loop iteration through experimental validation is required to progressively refine model predictivity. The deep integration of machine learning with experimental validation will provide an accelerating engine for the rational design of hydrogen spillover in NRA.

## Author contributions

Zhiwei Wang: writing – original draft. Junlong Zheng: data curation. Longchao Zhuo: review and editing. Xianghua Hou, Yinghong Wu, Wenxian Liu and Xijun Liu: supervision, resources, and conceptualization.

## Conflicts of interest

The authors declare that they have no known competing financial interests or personal relationships that could have appeared to influence the work reported in this paper.

## Data Availability

Data will be made available on request.
